# A nucleophilic beryllyl complex via metathesis at [Be–Be]^2+^

**DOI:** 10.1038/s41557-024-01534-9

**Published:** 2024-05-17

**Authors:** Josef T. Boronski, Agamemnon E. Crumpton, Aisling F. Roper, Simon Aldridge

**Affiliations:** Chemistry Research Laboratory Department of Chemistry, University of Oxford, Oxford, UK

**Keywords:** Chemical bonding, Chemical synthesis

## Abstract

Owing to its high toxicity, the chemistry of element number four, beryllium, is poorly understood. However, as the lightest elements provide the basis for fundamental models of chemical bonding, there is a need for greater insight into the properties of beryllium. In this context, the chemistry of the homo-elemental Be–Be bond is of fundamental interest. Here the ligand metathesis chemistry of diberyllocene (1; CpBeBeCp)—a stable complex with a Be–Be bond—has been investigated. These studies yield two complexes with Be–Be bonds: Cp*BeBeCp (2) and [K{(HCDippN)_2_BO}_2_]BeBeCp (3; Dipp = 2,6-diisopropylphenyl). Quantum chemical calculations indicate that the Be–Be bond in 3 is polarized to such an extent that the complex could be formulated as a mixed-oxidation state Be^0^/Be^II^ complex. Correspondingly, it is demonstrated that 3 can transfer the ‘beryllyl’ anion, [BeCp]^−^, to an organic substrate, by analogy with the reactivity of *sp*^2^–*sp*^3^ diboranes. Indeed, this work reveals striking similarities between the homo-elemental bonding linkages of beryllium and boron, despite the respective metallic and non-metallic natures of these elements.

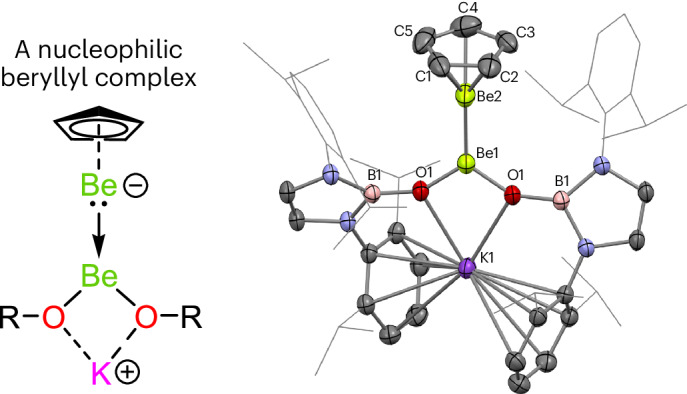

## Abstract (web summary)

The chemistry of beryllium, one of the periodic table’s lightest elements, remains poorly understood. Now, a nucleophilic beryllium complex with a highly polarized Be–Be bond has been prepared—this compound could be seen as a mixed-oxidation state Be(0)/Be(II) complex. The Be–Be bond is cleaved upon reaction with an organic substrate.

## Main

The properties of the very lightest elements (that is, those with principal quantum numbers of 1 or 2) form the basis for fundamental models of chemical bonding and reactivity^1^. It is notable, however, that the chemistry of the periodic table’s fourth element, beryllium, is very poorly developed owing to its extreme toxicity^2–5^. One essential aspect of the element’s properties that long remained unexplored in the condensed phases was the chemistry of the homo-elemental Be–Be bond^6–11^. In 1930, Herzberg unsuccessfully attempted to prepare diberyllium (Be_2_) in the gas phase; this diatomic species (with a formal bond order of zero) was first spectroscopically identified in 2009^12–14^. Moreover, despite numerous synthetic endeavours over the past 50 years, only in 2023 was the first example of a stable complex with a Be–Be bond—diberyllocene (CpBeBeCp, **1**)—structurally characterized^15–19^. Complex **1** is also a rare low-oxidation state beryllium species^9,20^. Indeed, several recent studies have reported low-coordinate beryllium complexes bearing redox non-innocent carbene ligands^21–26^. Although these have been formulated as ‘low valent’ beryllium complexes by some, such descriptions have been questioned by more recent theoretical studies^20,27–30^.

In contrast to the recent discovery of **1**, stable complexes with Mg–Mg bonds were first reported in 2007^31,32^. Attempts to prepare direct beryllium analogues of N-ligated magnesium(I) dimers were plagued by the formation of ligand- and solvent-activation products^15–19^. Also of relevance are diborane(4) compounds, which feature B–B bonds and are isoelectronic with **1** (ref. ^33^). The first diborane(4) derivative, B_2_Cl_4_, was prepared in 1925, and such species have subsequently found myriad applications in synthesis and catalysis. Quaternization of one of the boron centres of diborane(4) species by coordination of a nucleophile yields an *sp*^2^–*sp*^3^ diborane, with a polarized (*sp*^2^, *δ*^−^; *sp*^3^, *δ*^+^) B–B bond^34,35^. As such, these species have been used for the metal-free delivery of the boryl [BR_2_]^−^ anion to organic substrates.

In this Article, we investigate the metathesis chemistry of the [Be–Be]^2+^ core of **1** and report two complexes with Be–Be bonds: Cp*BeBeCp (**2**) and [K{(HCDippN)_2_BO}_2_]BeBeCp (**3**; Dipp = 2,6-diisopropylphenyl). These complexes are both unsymmetrical and, as a result, possess Be–Be bonds that are polarized, albeit to vastly differing degrees^34^. In the case of **3**, quantum chemical calculations suggest that the uneven distribution of electron density could be rationalized in terms of a mixed-valence Be^0^/Be^II^ formalism. This is supported by experimental studies, which reveal that **3** acts as a source of ‘beryllyl’ anions (formally featuring beryllium in the 0 oxidation state). Indeed, complex **3** is shown to transfer [BeCp]^−^ to carbon electrophiles in a manner analogous to boryl anion, [BR_2_]^−^, transfer by *sp*^2^–*sp*^3^ diboranes^33,34,36^.

## Results and discussion

### Metathesis reactions of diberyllocene

**Fig. 1:**
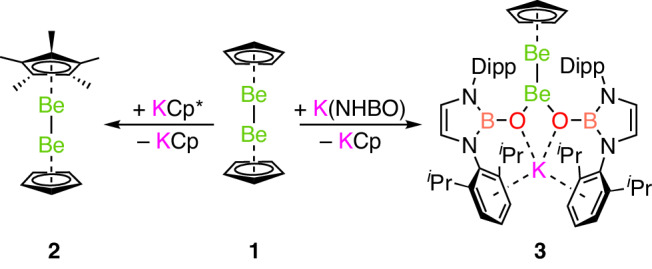
Synthesis of Cp*BeBeCp (2) and [K(NHBO)_2_]BeBeCp (3). Synthesis of complexes **2** and **3** via metathesis reactions with diberyllocene (**1**).

**Fig. 2:**
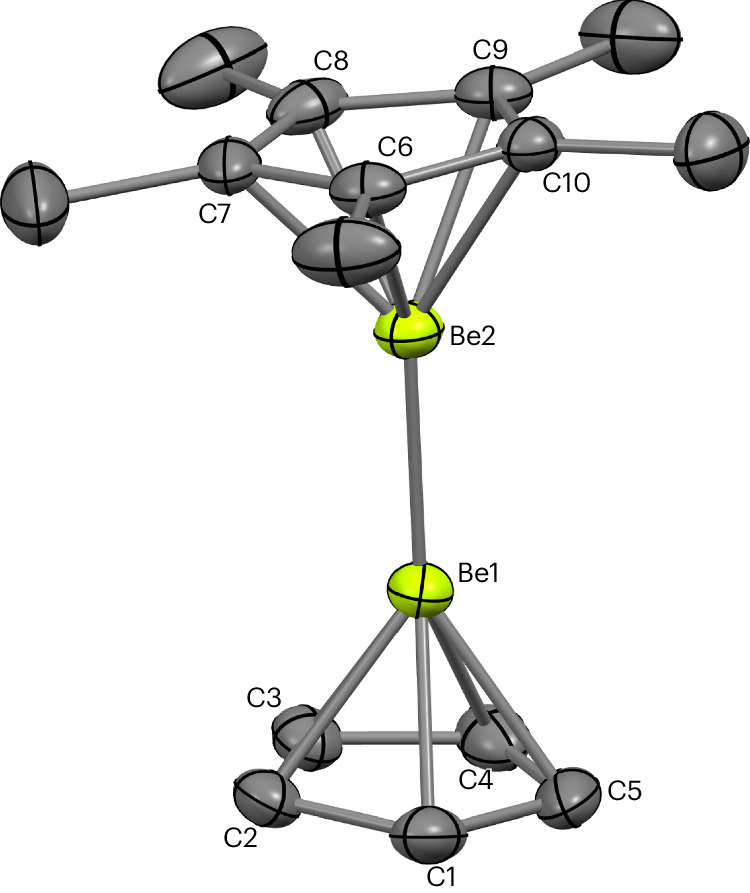
Molecular structure of complex 2. Molecular structure of complex **2** in the solid state, as determined by X-ray crystallography. Thermal ellipsoids set at 50% probability and hydrogen atoms omitted format for clarity.

**Fig. 3:**
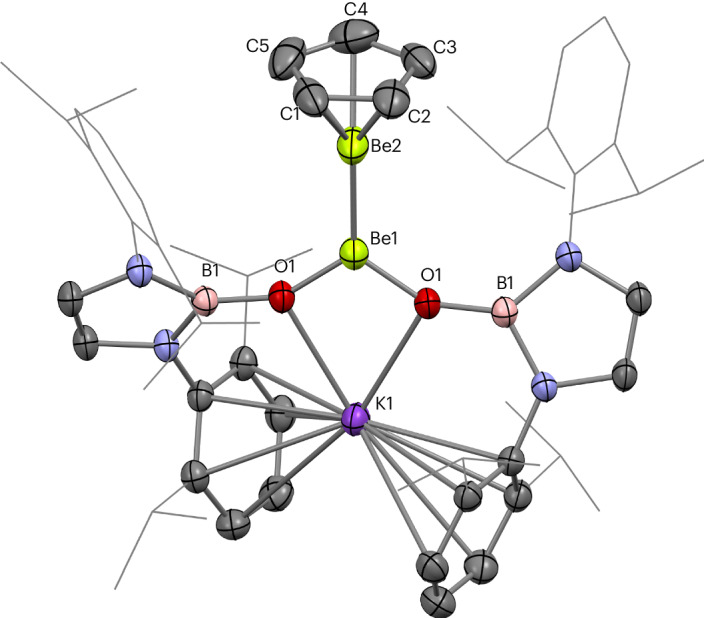
Molecular structure of 3. Molecular structure of complex **3** in the solid state, as determined by X-ray crystallography. Thermal ellipsoids set at 50% probability; selected substituents shown in wireframe format and hydrogen atoms omitted for clarity.

Diberyllocene (**1**) and decamethyldizincocene, Cp*ZnZnCp* (**A**), are both calculated to feature robust homometallic bonds (homolytic bond dissociation energy for CpMMCp: M = Be, 71.7 kJ mol^−^^1^; M = Zn, 70.3 kJ mol^−^^1^)^6,10,37^. Indeed, **A** has been found to participate in ligand metathesis reactions, which leave the Zn–Zn bond intact^38–43^. Thus, we decided to investigate whether a salt metathesis approach would enable the substitution of the cyclopentadienyl ligands of **1** (refs. ^18,44^). Heating **1** and an excess of KCp* in benzene for 4 days leads to the quantitative formation of Cp*BeBeCp (**2**) and KCp (Fig. 1); no evidence for further substitution of the remaining Cp ligand was obtained even under forcing conditions. By contrast, reaction of **1** with two equivalents of the potassium salt of the N-heterocyclic boryloxy (NHBO) ligand K[(HCDippN)_2_BO] is rapid, yielding [K(NHBO)_2_]BeBeCp (**3**), again with the concomitant formation of KCp^45^ (Fig. 1). Both **2** and **3** could be crystallized from hexane and their molecular structures determined by single-crystal X-ray diffraction (SCXRD) (Figs. 2 and 3, respectively).

Complex **2**, like **1**, is a dimetallocene, consisting of two beryllium half-sandwich units—in this case one BeCp* and one BeCp—linked by a Be–Be bond^37^. There are two Cp*BeBeCp molecules in the asymmetric unit of **2**. Of these two independent molecules, one exhibits a slightly staggered conformation of cyclopentadienyl ligands, whereas in the other complex, these carbocyclic ligands are almost perfectly eclipsed, as they are in **1**. The Be–Be distances measured for the two independent Cp*BeBeCp units are 2.065(3) Å and 2.045(3) Å, which are statistically indistinguishable from the Be–Be bond of **1** (2.0545(18) Å). Unlike in **1**, the orientations of the cyclopentadienyl ligands of the two molecules of **2** deviate somewhat from a parallel alignment, with interplanar angles of 8.71(9)° and 12.84(10)° (ref. ^46^). In addition, the Be–Cp_cent_ distances are 1.528(4) Å and 1.521(4) Å, respectively, and the Be–Cp*_cent_ distances are 1.501(4) Å and 1.491(5) Å, respectively. Hence, the Be–Cp_cent_ distances in **2** are comparable to the analogous metric measured for **1** (1.519(3) Å)^18^.

Complex **3** also features a BeCp unit, in addition to a [K(NHBO)_2_]Be moiety, with two NHBO ligands coordinated to one Be centre through Be–O bonds^45^. The two beryllium centres of **3** are connected to one another via a Be–Be bond. The two NHBO ligands of **3** also coordinate a potassium cation via K···(η^6^-C_6_H_3_^*i*^Pr_2_) and K···O interactions, thereby forming a four-membered BeO_2_K ring. The Be1–Be2 distance within **3** (2.135(3) Å) is markedly longer than those measured for **1** and **2** (2.0545(18) Å and 2.045(3)/2.065(3) Å, respectively)^18^. In addition, the Be–Cp_cent_ distance measured for **3** (1.578(2) Å) is considerably longer than the corresponding metrics for **1** and **2** (1.519(3) Å and 1.528(4) Å, respectively), suggesting that Be2 in **3** is highly electron rich. The Be1–O1 and K1···O1 distances are 1.5639(14) Å and 2.6561(8) Å, respectively. This Be–O distance is somewhat longer than those reported for the three-coordinate beryllium(II) aryloxide complexes Be(ODipp)_2_(SIPr) (1.497(3)/1.507(3) Å; SIPr = 1,3-diisopropyl-4,5-dimethylimidizol-2-ylidene) and Be(OMes*)_2_(Et_2_O) (1.481(2) Å; Mes* = 2,4,6-^*t*^Bu_3_C_6_H_2_), as well as for the homoleptic two-coordinate NHBO complex Be(NHBO)_2_ (**4**; 1.4266(8) Å; vide infra), probably owing to the greater ionic radius of Be^I^ compared with Be^II^ (refs. ^18,47,48^). The O1–Be1–O1′ angle in **3** is 107.88(7)° and the sum of the angles at Be1 is 360.00(16)°, consistent with a trigonal planar geometry for this beryllium centre. This mirrors the trigonal planar geometry of the boron centres within diborane(4) derivatives^33^.

It should be noted that ligand metathesis at a [Mg–Mg]^2+^ unit has not previously been reported^32^. Moreover, all structurally characterized magnesium(I) dimers feature supporting ligands with N-donor groups. Juxtaposing this, a variety of supporting ligands have been found to be suitable for stabilizing the Zn–Zn bond, including halides, aryloxides and even simple Lewis bases (for example, 4-dimethylaminopyridine and tetrahydrofuran)^38–41,43^. The lability of **1** with respect to ligand metathesis (that is, the retention of the homometallic linkage) provides further experimental evidence for the theoretically predicted chemical similarities between the [Zn–Zn]^2+^ and [Be–Be]^2+^ moieties^6,10^.

Complexes **2** and **3** were characterized by multinuclear NMR spectroscopy. Perhaps most informative are the ^9^Be NMR chemical shifts measured for these complexes^49,50^. The ^9^Be NMR spectrum of complex **2** features resonances at −28.6 ppm and −21.7 ppm, attributable to the Cp-coordinated beryllium centre (Be_Cp_) and Cp*-coordinated beryllium centre (Be_Cp*_), respectively. These ^9^Be NMR chemical shifts are very similar to those measured for the Be centres of **1** and BeCp*_2_ (−27.6 ppm and −21.7 ppm, respectively)^18,51^. By contrast, in the case of **3**, disparate ^9^Be NMR resonances are observed at −29.8 ppm and +9.5 ppm, which correspond to Be_Cp_ and the NHBO-coordinated beryllium centre (Be_NHBO_), respectively. The upfield resonance has the lowest chemical shift of any reported ^9^Be NMR signal and indicates that Be_Cp_ is a highly electron-rich low-oxidation state beryllium centre^18,44,49,50^ (Supplementary Table 2 and Supplementary Fig. 20). The ^9^Be NMR resonance corresponding to Be_NHBO_ is similar to that measured for the beryllium(II) complex Be(NHBO)_2_ (**4**, +5.1 ppm; vide infra) and is extremely broad (full-width at half-maximum (*w*_1/2_) 380 Hz), as is typically found for low-coordinate, electron-poor beryllium centres^49^. For comparison, the *w*_1/2_ of the ^9^Be resonance measured for **1** is 33.4 Hz (ref. ^18^). No evidence of measurable Be–Be coupling could be observed in the ^9^Be NMR spectra of either **2** or **3**.

### Computational investigations of relevant complexes

**Fig. 4:**
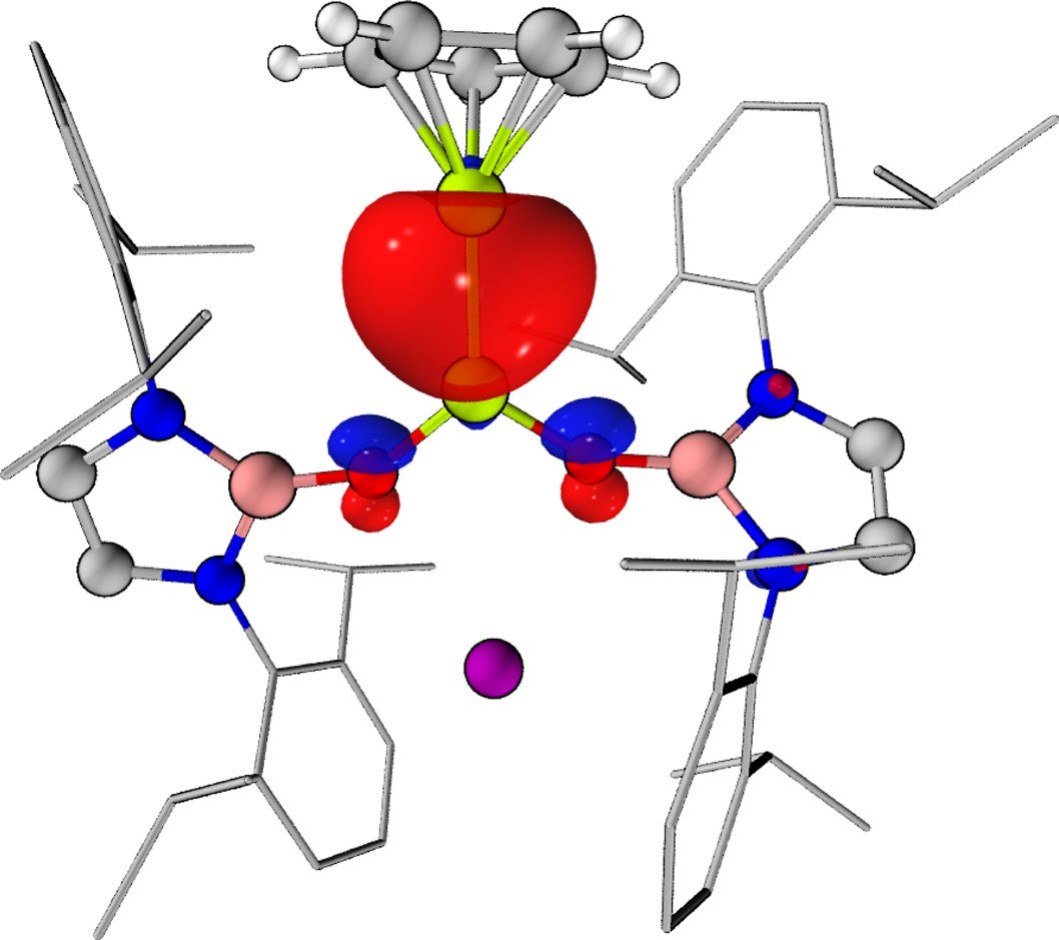
HOMO of compound 3. HOMO of complex **3**, corresponding to the principal Be–Be bonding interaction (0.05 a.u.). Complex geometry optimized with the r^2^SCAN-3c composite method, followed by a single-point calculation using the ωB97X-D4 functional with the def2-QZVPP basis set.

To gain further insight into the electronic structure and bonding of **2** and **3**, both complexes were investigated using quantum chemical calculations. The structures of complexes **2** and **3** (and, for comparative purposes, **1**) were optimized with the r^2^SCAN-3c composite method, and a single-point calculation was performed on this optimized geometry using the ωB97X-D4 functional with the def2-QZVPP basis set. The highest occupied molecular orbital (HOMO) of all three complexes corresponds to a Be–Be bonding orbital of *σ*-symmetry^18^. In the case of **1** and **2**, the HOMO is distributed equally between both beryllium centres (Supplementary Figs. 21 and 22). However, the HOMO of complex **3** has a hemispherical profile, with a greater proportion of the electron density localized on Be_Cp_ than on Be_NHBO_, thus indicating that the Be–Be interaction in this complex is polarized^1^ (Fig. 4).

To provide further evidence for this observation, quantum theory of atoms in molecules (QTAIM) and electron localization function (ELF) calculations were performed on **1**, **2** and **3**. In the case of **1** and **2**, QTAIM calculations find a (3, −3) critical point, or a non-nuclear attractor (NNA), in addition to two (3, −1) bond critical points, for the Be–Be interactions in both complexes (Supplementary Figs. 28 and 29). This is consistent with previous calculations on **1** and with experimental and theoretical studies of magnesium(I) dimers^10,18,52^. By contrast, complex **3** is not calculated to feature an NNA, indicating fundamental differences between the Be–Be interaction in this complex and that in **1** and **2** (ref. ^53^) (Supplementary Fig. 30). The Bader charges calculated for the Be centres within **1** (+1.42) and **2** (Be_Cp_, +1.39; Be_Cp*_, +1.43) are very similar, with the NNA bearing a large negative charge in both cases (**1**, −1.17; **2**, −1.15). Indeed, the Be–Be bond of **2** is clearly only very slightly polarized. In the case of **3**, however, the charge distribution at the beryllium centres is highly uneven (Be_Cp_, +0.19; Be_NHBO_, +1.62). The Bader charge for Be_Cp_ in **3** implies that this beryllium centre is extremely electron rich and is consistent with the upfield ^9^Be NMR resonance and long Be–Cp_cent_ distance measured for this complex^54,55^. This charge distribution also aligns with the composition of the ELF basin associated with the Be–Be interaction in **3**, which features a much greater contribution from Be_Cp_ (1.67 *e*^−^) than the Be_NHBO_ (0.24 *e*^−^)^56,57^. Combined, these data imply that complex **3** could be considered a mixed-valence Be_Cp_^0^/Be_NHBO_^II^ complex with a Be^0^ → Be^II^ donor–acceptor bond^56,57^.

Natural bond orbital (NBO) and natural population analysis (NPA) calculations also indicate that the Be–Be interaction in **3** is highly polarized; NPA charges of +0.64 for Be_Cp_ and +1.15 for Be_NHBO_ were calculated (Supplementary Figs. 33–35 and Supplementary Tables 6 and 7). In a similar fashion to the ELF calculations, NBO analysis indicates starkly different contributions to the Be–Be interaction from the two beryllium centres in **3**, with 62% of the electron density donated by Be_Cp_ and 38% by Be_NHBO_^56,57^. The populations of the valence 2*s* and 2*p* orbitals of the two beryllium centres also evidence the greater degree of electron density at Be_Cp_ (1.34 *e*^−^) compared with Be_NHBO_ (0.85 *e*^−^).

**Fig. 5:**
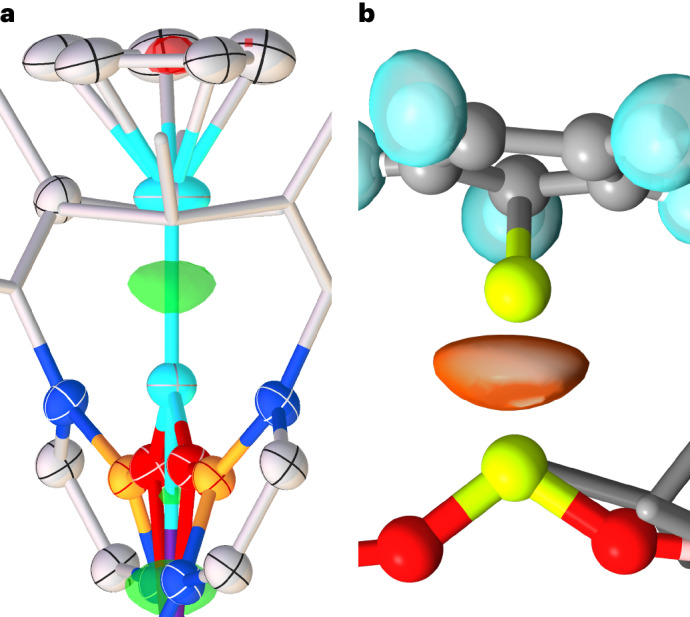
Experimental and computational analysis of Be–Be bonding in complex 3. **a**, The SCXRD-derived residual electron density plot for complex **3**. **b**, ELF isosurface for the Be–Be interaction in **3** (right; orange; 0.7 a.u.).

To obtain further evidence for the polarized nature of the Be–Be interaction in **3**, the SCXRD-derived residual electron density map for this complex (refined using non-spherical atomic form factors) was examined^52,58,59^. There is a resemblance between the residual electron density in this plot and the profile of the ELF isosurface corresponding to the Be–Be bonding basin (Fig. 5). Indeed, in both cases, the electron density appears to be polarized, with a hemispherical lobe directed from Be_Cp_ towards Be_NHBO_. To benchmark the ELF data for **3**, the isosurfaces corresponding to the B–B bond of *sp*^2^–*sp*^3^ diborane [(pin)B–B(F)(pin)]^−^ (**B**; pin = [{OC(Me)_2_}_2_]^2^^−^) and the archetypal donor–acceptor N–B bond of H_3_NBH_3_ (**C**) were plotted using the same method^35^ (Supplementary Figs. 36 and 37). Visually, these two ELF isosurfaces, and that of the Be–Be interaction in **3**, have similar hemispherical profiles, with the electron density directed from the Lewis basic moiety towards the Lewis acidic centre. This topology is typical of the forms of ELF isosurfaces for donor–acceptor interactions^56–58^. It should be noted that the ELF isosurfaces for the Be–Be interaction **1** and **2** (as well as a range of polarized and non-polarized C–C bonds) have symmetrical, pancake-like profiles, which are typical of covalent-type bonds and are therefore markedly different from that of **3** (ref. ^58^) (Supplementary Figs. 31, 32 and 38–40).

To further examine the nature of the Be–Be interaction in **3** computationally, energy decomposition analysis (EDA) calculations were performed on this complex, as well as **1**, **2**, **B** and **C**. It has previously been found (by ourselves and others) that natural energy decomposition analysis (NEDA) is effective for probing the nature of bonding in main group element-containing molecules; thus, we used this technique here^60,61^ (Supplementary Tables 8–12). The most representative bonding model for a given molecule can be determined by the fragments that yield the lowest magnitude of the orbital interaction energy (*E*_orb_) upon recombination^20,27,28,62–64^. Indeed, in the case of **3**, |*E*_orb_| for a donor–acceptor recombination ([CpBe] → [Be(NHBO)_2_K], −217 kcal mol^−^^1^) is lower than that of the (homolytic) radical recombination of the Be–Be linkage (−282 kcal mol^−^^1^). This mirrors calculations performed on **B** and **C**, for which |*E*_orb_| for the donor–acceptor recombination ([(pin)B → B(F)(pin)]^−^, −411 kcal mol^−^^1^; H_3_N → BH_3_, −216 kcal mol^−^^1^) is lower, in each case, than the respective homolytic radical recombinations (−521 kcal mol^−^^1^ and −532 kcal mol^−^^1^, respectively). This contrasts with the corresponding analysis on **2**, for which a homolytic radical Be–Be recombination is favoured (*E*_orb_ = −224 kcal mol^−^^1^), compared with both donor–acceptor formulations for the complex ([CpBe] → [BeCp*], −275 kcal mol^−^^1^; [Cp*Be] → [BeCp], −276 kcal mol^−^^1^). Hence, NEDA also indicates that a donor–acceptor, Be^0^/Be^II^ formalism could be used to describe the electronic configuration of **3** (ref. ^27^).

EDA coupled with natural orbitals for chemical valence (EDA–NOCV) calculations were also performed on **1**, **2** and **3** (ref. ^1^). The |*E*_orb_| for the (homolytic) biradical fragmentation of the Be–Be linkage of **3** (−70.1 kcal mol^−^^1^; Supplementary Figs. 47 and 48) is lower than the corresponding |*E*_orb_| for a pure donor–acceptor fragmentation ([CpBe] → [Be(NHBO)_2_K], −116 kcal mol^−^^1^; Supplementary Fig. 49). Nonetheless, in the case of homolytic fragmentation, the eigenvalues for the *α*_1_- and *β*_1_-pair densities are highly dissimilar ([CpBe] → [Be(NHBO)_2_K], 0.36; [CpBe] ← [Be(NHBO)_2_K], 0.29), thereby indicating that there is a large net movement of electrons from Be_Cp_ to Be_NHBO_ (Supplementary Table 15). Again, this evidences the highly polarized nature of the Be–Be linkage in **3**. For reference, EDA–NOCV calculations were also performed on a range of other molecules with polar and non-polar homo-elemental (B–B and C–C) bonds (Supplementary Tables 14–16 and Supplementary Figs. 50–61). Of all these examples, homolytic fragmentation of the Be–Be bond of **3** was calculated to lead to the greatest net movement of electrons. Thus, the Be–Be linkage of **3** could be described as the most polarized bond of all those studied here.

When complexes of the form [Be(cAAC)_2_] (**D**; cAAC = cyclic(alkyl)(amino)carbene) were initially reported, they were described as comprising a neutral beryllium(0) centre and two neutral carbene ligands^21^. However, cAAC ligands are known to be redox non-innocent, and several subsequent theoretical studies indicate that **D** is instead best described as a beryllium(II) complex featuring two anionic cAAC ligands^20,29^. Indeed, this is consistent with our own calculations on **D**, which yield Bader and NPA charges of +1.50 and +1.47, respectively, for the beryllium centre within this complex. These values are much greater than the same metrics calculated for Be_Cp_ in **3** (+0.19 and +0.64, respectively). In broader terms, the formal oxidation state assignment for a particular element centre is only meaningful in the context of its chemical behaviour. Indeed, although **D** has been used as a reducing agent, this reactivity could be ascribed to the anionic nature of the carbene ligands within this complex^25^. As far as we are aware, there are no reported data that unequivocally show that **D** reacts as a source of low-valent beryllium.

### Reactivity studies of complex 3

**Fig. 6:**
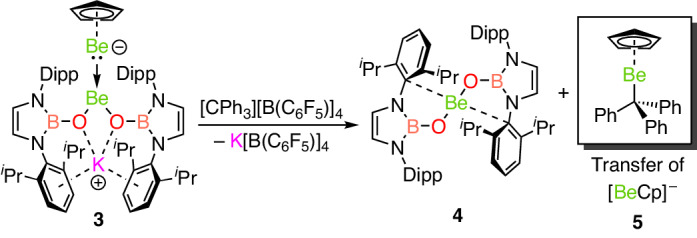
Transfer of the beryllyl anion, [BeCp]^−^, to an organic substrate by complex 3. Reactivity of complex **3** with [CPh_3_][B(C_6_F_5_)_4_], a source of the organic electrophile [CPh_3_]^+^.

To obtain experimental evidence for the possible Be^0^/Be^II^ formulation for **3**, the reactivity of this complex was examined. In this context, the reaction of **3** with [CPh_3_][B(C_6_F_5_)_4_] is informative: the process leads to the formation of previously unreported complexes Be(NHBO)_2_ (**4**; Supplementary Fig. 15) and CpBe(CPh_3_) (**5**; Supplementary Fig. 16), as well as K[B(C_6_F_5_)_4_] (Fig. 6). This reaction involves the transfer of a nucleophilic ‘beryllyl’ anion, [CpBe]^−^ (which features a formal beryllium(0) centre), from **3** to the electrophilic trityl cation, forming a Be–C bond and yielding **5**, leaving the beryllium(II)-containing fragment **4** (ref. ^44^). This reactivity is analogous to that of *sp*^2^–*sp*^3^ diboranes, which feature highly polarized B–B bonds and act as a source of the boryl anion, [BR_2_]^−^, which is closely related to the beryllyl anion^33–36^. As such, the observed reactivity of **3** provides evidence that this species could be considered a mixed-oxidation state Be^0^/Be^II^ complex, and is a rare example of a nucleophilic *s*-block complex.

## Conclusion

We have prepared two complexes with Be–Be bonds through ligand metathesis reactions of diberyllocene (CpBeBeCp, **1**). These complexes—Cp*BeBeCp (**2**) and [K{(HCDippN)_2_BO}_2_]BeBeCp (**3**)—have been studied by quantum chemical methods, which imply that **3** could be formulated as a Be^0^/Be^II^ complex. This assignment is supported by quantum chemical calculations and residual electron density plots, which are derived from SCXRD measurements. Further experimental evidence for this claim has been obtained through transfer of the beryllyl anion, [CpBe]^−^, to an electrophilic organic substrate and isolation of both beryllium-containing products. Fundamentally, this reactivity can be likened to that of *sp*^2^–*sp*^3^ diboranes, revealing that the reactivities of homo-elemental bonds of beryllium and boron have parallels, despite the respective metallic and non-metallic natures of these elements. Hence, this work reveals that bonding trends across period 2 are more continuous than may previously have been appreciated. We continue to investigate the chemical relationships between beryllium and its neighbouring elements to test models of chemical bonding.

## Methods

### General considerations

Beryllium and its compounds are extremely toxic and can cause irreversible health effects through inhalation or skin contact. The work with beryllium-containing materials described herein was carried out by trained operators, with strict adherence to local and national rules and regulations^5^.

All manipulations were carried out using Schlenk line or glovebox techniques under an atmosphere of argon or dinitrogen. Solvents were dried by passage through activated alumina towers, dried with NaK_2_ and degassed before use. Solvents were stored over NaK_2_. NMR spectra were measured in C_6_D_6_, which was dried over NaK_2_, with the solvent being distilled under reduced pressure, degassed by three freeze–pump–thaw cycles and stored under argon in a Teflon valve ampoule. NMR samples were prepared under argon in 5 mm Wilmad 507-PP tubes fitted with J. Young Teflon valves. NMR spectra were measured on a Bruker Avance III HD Nanobay 400 MHz NMR spectrometer equipped with a 9.4 T magnet or a Bruker Avance III 500 MHz NMR spectrometer equipped with an 11.75 T magnet. ^1^H and ^13^C NMR spectra were referenced internally to residual protio-solvent (^1^H) or solvent (^13^C) resonances and are reported relative to tetramethylsilane (*δ* = 0 ppm). ^9^Be NMR spectra were referenced to a 0.43 M solution of BeSO_4_·4H_2_O in D_2_O (*δ* = 0 ppm). Chemical shifts are quoted in *δ* (ppm) and coupling constants in Hz. The compounds diberyllocene (**1**), [(HCDippN)_2_BO]K (K-NHBO), KCp* and [CPh_3_][B(C_6_F_5_)_4_] were prepared as described previously^18^^,^^45^^,^^65^.

### Synthesis of novel compounds

#### Synthesis of Cp*BeBeCp (2)

A solid mixture of **1** (4.0 mg, 0.027 mmol) and KCp* (19 mg, 0.11 mmol, 4.0 equiv.) was added to an ampoule fitted with a Teflon valve and equipped with a glass-coated stirrer bar. Benzene (1 ml) was condensed into the vessel in vacuo at −196 °C. The colourless suspension was allowed to warm to room temperature and stirred at 80 °C for 4 days. Volatiles were removed in vacuo at 0 °C, leaving a white powder. Complex **2** is volatile, so prolonged drying should be avoided. Soluble material was dissolved in hexane (0.5 ml), filtered and concentrated (0.2 ml). Concentration of a hexane solution in a sealed glass λ-type tube resulted in the formation of colourless crystals, which were carefully dried in vacuo. Yield: 4.0 mg, 68%. Single crystals of **2** suitable for X-ray diffraction experiments were obtained by slow concentration of a hexane solution in a sealed glass λ-type tube. Analytically calculated for C_15_H_20_Be_2_: C, 82.51; H, 9.23. Found: C, 81.96; H, 9.16. ^1^H NMR (400 MHz, C_6_D_6_, 298 K): *δ* = 1.90 (s, 15H, C_5_(C*H*_3_)_5_), 5.68 (s, 5H, C_5_*H*_5_); ^9^Be NMR (42 MHz, C_6_D_6_): *δ* = −28.6 (*w*_1/2_ = 49.3 Hz; Cp*Be*), −21.7 (*w*_1/2_ = 39.9 Hz; Cp*Be); ^13^C{^1^H} NMR (101 MHz, C_6_D_6_): *δ* = 10.3 (C_5_(*C*H_3_)_5_), 102.3 (*C*_5_H_5_), 108.2 (*C*_5_(CH_3_)_5_).

#### Synthesis of [K{(HCDippN)_2_BO}_2_]BeBeCp (3)

A solid mixture of **1** (4.0 mg, 0.027 mmol) and K-NHBO (23 mg, 0.054 mmol, 2.0 equiv.) was added to an ampoule fitted with a Teflon valve and equipped with a glass-coated stirrer bar. Benzene (1 ml) was condensed into the vessel in vacuo at −196 °C. The pale-yellow suspension was allowed to warm to room temperature and stirred for 1 h. Subsequently, volatiles were removed in vacuo, and soluble material was dissolved in hexane (0.5 ml). The suspension was filtered and concentrated (0.2 ml). Concentration of a hexane solution in a sealed glass λ-type tube resulted in the formation of colourless crystals, which were carefully dried in vacuo. Yield: 18 mg, 73%. Single crystals of **3** suitable for X-ray diffraction experiments were obtained by slow concentration of a hexane solution in a sealed glass λ-type tube. Analytically calculated for C_57_H_77_B_2_Be_2_KN_4_O_2_: C, 73.69; H, 8.35; N, 6.03. Found: C, 73.32; H, 8.11; N, 5.85. ^1^H NMR (400 MHz, C_6_D_6_, 298 K): *δ* = 1.20 (2 x d, 48H, CH(C*H*_3_)_2_), 3.49 (sept, ^3^*J*_HH_ = 6.8 Hz, 8H, C*H*(CH_3_)_2_), 5.49 (s, 5H, C_5_*H*_5_), 5.93 (s, 4H, C*H*), 7.06 (m, 12H, Ar*H*); ^9^Be NMR (42 MHz, C_6_D_6_): *δ* = −29.8 (*w*_1/2_ = 94.7 Hz; Cp*Be*), 9.5 (*w*_1/2_ = 380 Hz; O_2_*Be*); ^11^B NMR (128 MHz, C_6_D_6_): *δ* = 20.5; ^13^C{^1^H} NMR (101 MHz, C_6_D_6_): *δ* = 23.7 (CH(*C*H_3_)_2_), 24.6 (CH(*C*H_3_)_2_), 28.3 (*C*H(CH_3_)_2_), 102.7 (*C*_5_H_5_), 116.5 (N*C*H), 123.7 (Dipp-*m*-*C*H), 126.4 (Dipp-*p*-*C*H), 141.9 (Dipp-*i*-*C*), 147.4 (Dipp-*o*-*C*).

#### Synthesis of Be(NHBO)_2_ (4) and CpBe(CPh_3_) (5)

A solid mixture of **3** (8.0 mg, 0.0086 mmol) and [CPh_3_][B(C_6_F_5_)_4_] (7.9 mg, 0.0086 mmol) was added to an ampoule fitted with a Teflon valve and equipped with a glass-coated stirrer bar. Benzene (1 ml) was condensed into the vessel in vacuo at −196 °C. The yellow solution was allowed to warm to room temperature and stirred for 1 h. Subsequently, volatiles were removed in vacuo, soluble material was dissolved in hexane (0.5 ml), and the suspension was filtered. Slow concentration of the resulting solution in a sealed glass λ-type tube led to the formation of colourless crystals (**4**) and yellow crystals (**5**), which were manually separated. Compounds **4** and **5** degrade rapidly in the solid state and in solution. Yield of **4**: 3.8 mg, 54%. Yield of **5**: 2.1 mg, 76%. Compound **4**: analytically calculated for C_52_H_72_B_2_BeN_4_O_2_: C, 76.56; H, 8.90; N, 6.87. Found: C, 76.39; H, 8.86; N, 6.74. ^1^H NMR (400 MHz, C_6_D_6_, 298 K): *δ* = 1.04 (d, ^3^*J*_HH_ = 7.1 Hz, 24H, CH(C*H*_3_)_2_),1.19 (d, ^3^*J*_HH_ = 6.8 Hz, 24H, CH(C*H*_3_)_2_), 3.16 (sept, ^3^*J*_HH_ = 7.5 Hz, 8H, C*H*(CH_3_)_2_), 5.86 (s, 4H, C*H*), 7.09 (m, 12H, Ar*H*); ^9^Be NMR (42 MHz, C_6_D_6_): *δ* = 5.1 (*w*_1/2_ = 727.4 Hz); ^11^B NMR (128 MHz, C_6_D_6_): *δ* = 20.6; ^13^C{^1^H} NMR (101 MHz, C_6_D_6_): *δ* = 24.0 (CH(*C*H_3_)_2_), 24.4 (CH(*C*H_3_)_2_), 28.6 (*C*H(CH_3_)_2_), 116.0 (N*C*H), 123.5 (Dipp-*m*-*C*H), 127.4 (Dipp-*p*-*C*H), 137.8 (Dipp-*i*-*C*), 146.8 (Dipp-*o*-*C*). Compound **5**: analytically calculated for C_24_H_20_Be: C, 90.81; H, 6.35. Found: C, 90.48; H, 6.32. ^1^H NMR (400 MHz, C_6_D_6_, 298 K): *δ* = 5.61 (s, 5H, C_5_*H*_5_), 7.03 (t, ^3^*J*_HH_ = 7.1, 6H, *o*-C_6_*H*_5_), 7.10 (t, ^3^*J*_HH_ = 7.3, 6H, *m*-C_6_*H*_5_), 7.36 (d, ^3^*J*_HH_ = 7.6, 3H, *p*-C_6_*H*_5_); ^9^Be NMR (42 MHz, C_6_D_6_): *δ* = −18.0 (*w*_1/2_ = 29.4 Hz); ^13^C{^1^H} NMR (101 MHz, C_6_D_6_): 102.7 (*C*_5_H_5_), 131.0 (*C*_6_H_5_), 137.9 (*C*_6_H_5_), 141.5 (*C*_6_H_5_), 145.0 (*C*_6_H_5_), 187.3 (*C*Ph_3_).

### Crystallographic data

Crystallographic data for **2**–**5** were collected using an Oxford Diffraction (Agilent) SuperNova or Rigaku XtaLAB Synergy-R. Crystals were selected under Paratone-N or perfluorinated oil, mounted on MiTeGen Micromount loops and quench-cooled using an Oxford Cryosystems open flow N_2_ cooling device^66^. Selected details of data collection are given in Supplementary Table 1. Data collected were processed using the CrysAlisPro package, including unit cell parameter refinement and inter-frame scaling (which was carried out using SCALE3 ABSPACK within CrysAlisPro)^67^. Equivalent reflections were merged and diffraction patterns processed with the CrysAlisPro suite. Structures were solved ab initio from the integrated intensities using SHELXT and refined on F2 using SHELXL with the graphical interface OLEX2^68–70^. In the case of complex **3**, the NoSpherA2 method was used for refinement using non-spherical form factors^59,71^. In this case, r^2^SCAN def2-SVP was used in the iterative improvement of the fit of the model (followed by a single-point calculation; r^2^SCAN def2-TZVP) using ORCA version 5.0.4 (refs. ^72–76^). Crystallographic data are given in the supplementary deposited CIF files (CCDC 2324714–2324717) and can be obtained free of charge from the Cambridge Crystallographic Data Centre via http://www.ccdc.cam.ac.uk/data_request/cif. Structures of **3** and **5** feature disorder of the cyclopentadienyl groups over special positions. This was treated using part −1 and fractional occupancy in both cases. Further details of how crystallographic disorder was treated can be found in the CIF files.

### Computational details

The structures of complexes **1**–**3**, **3′** (compound **3** without potassium cation [{(HCDippN)_2_BO}_2_BeBeCp]^−^) and **B**–**D** were optimized using ORCA (revision 5.0.4)^72,73^. Specifically, r^2^SCAN was used^74,77^, in conjunction with the def2-TZVPPm basis set with the D4 dispersion correction^78^, using the geometrical counterpoise correction gCP (together known as the r^2^SCAN-3c method) and CPCM solvent (benzene) modelling^79,80^. Subsequently, a single-point calculation was performed on this optimized structure with the ωB97X range-separated hybrid functional^75,81^, in conjunction with the def2-QZVPP basis set^76,82^ and the D4 dispersion correction^78^. The nature of the stationary points (minima) was confirmed by full frequency calculations, which are characterized by zero imaginary frequencies. NBO calculation was performed on the ORCA wavefunction using NBO 7.0 (ref. ^83^). QTAIM calculations were performed using the ORCA wavefunctions for the respective complexes and were generated using Multiwfn 3.8 and AIMALL^84,85^. ELF calculations were performed using the ORCA wavefunctions for the respective complexes, generated using Multiwfn 3.8 (ref. ^86^). All EDA was performed using Gaussian 16 (revision C.01) at the M062x-def2-TZVP level^87^.

## Online content

Any methods, additional references, Nature Portfolio reporting summaries, source data, extended data, supplementary information, acknowledgements, peer review information; details of author contributions and competing interests; and statements of data and code availability are available at 10.1038/s41557-024-01534-9.

### Supplementary information

Supplementary Information Supplementary Figs. 1–61 and Tables 1–16.

Supplementary Data 1 Cartesian coordinates of the optimized structures.

Supplementary Data 2 Crystallographic data for compound **2**; CCDC reference no. 2324716.

Supplementary Data 3 Crystallographic data for compound **3**; CCDC reference no. 2324715.

Supplementary Data 4 Crystallographic data for compound **4**; CCDC reference no. 2324717.

Supplementary Data 5 Crystallographic data for compound **5**; CCDC reference no. 2324714.

## Data Availability

All data generated or analysed during this study are included in this published article (and its Supplementary Information files). X-ray data are available free of charge from the Cambridge Crystallographic Data Centre (CCDC 2324714 (**5**), 2324715 (**3**), 2324716 (**2**) and 2324717 (**4**)). Cartesian coordinates of optimized structures are available as a Supplementary Information file.
