# Supplementary material for: A nucleophilic beryllyl complex via metathesis at [Be–Be]2+
Source: Nat Chem. 2024 May 17;16(8):1295–300. doi: 10.1038/s41557-024-01534-9 (PMC11321998; doi:10.1038/s41557-024-01534-9)
Supplement: Supplementary file 1 — Supplementary Figs. 1–61 and Tables 1–16. [file 41557_2024_1534_MOESM1_ESM.pdf]

nature chemistry

Article

https://doi.org/10.1038/s41557-024-01534-9

A nucleophilic beryllyl complex via metathesis at $[Be–Be]^{2+}$

In the format provided by the authors and unedited

Contents

Spectroscopic Data – S2

Crystallographic Data – S11

Discussion of ^{9}Be NMR Chemical Shifts – S13

Computational Details – S15

Relevant molecular orbitals – S15

Quantum Theory of Atoms in Molecules Calculations – S18

Electron Localisation Function Isosurfaces and Residual Electron Density Maps – S22

Natural Bond Orbital and Natural Population Analysis Calculations – S27

Natural Energy Decomposition Analysis Calculations – S29

Energy Decomposition Analysis - NOCV – S30

References – S38

Spectroscopic Data

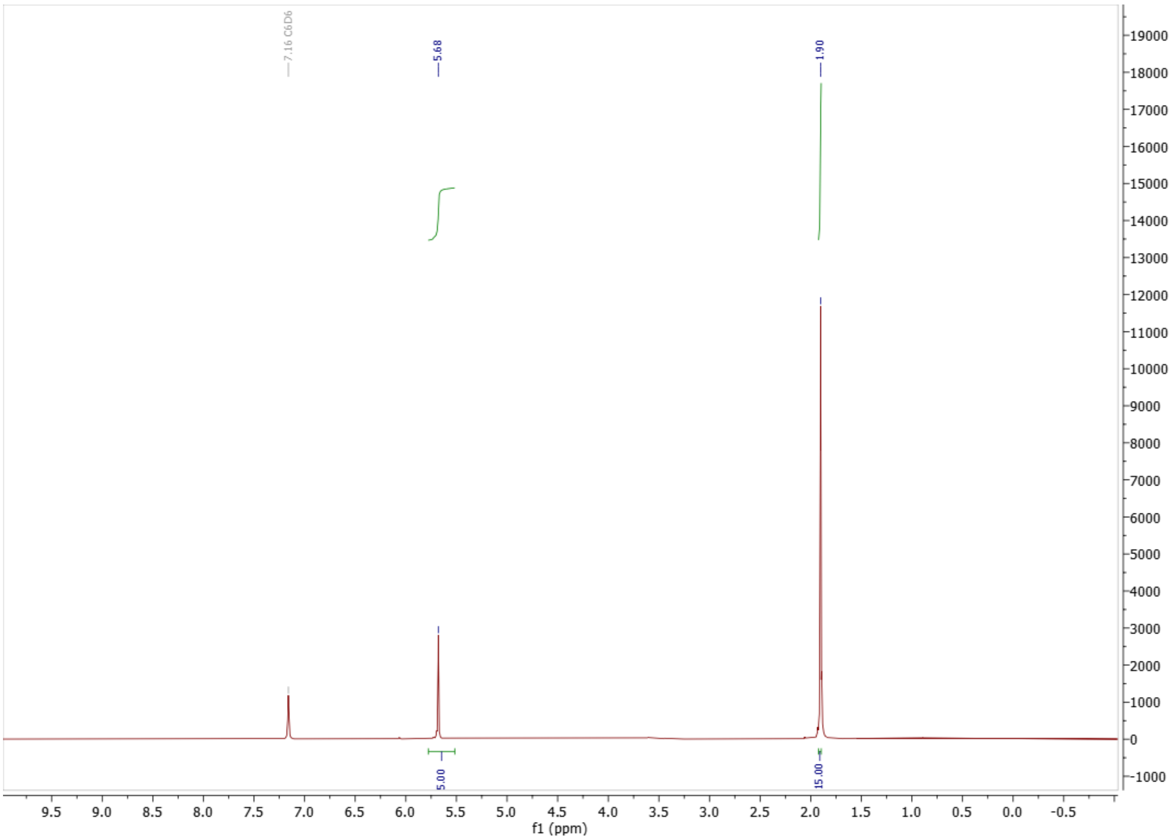

Supplementary Figure 1: ^{1}H NMR (400 MHz) spectrum of compound **2** in C_6D_6 at 298 K.

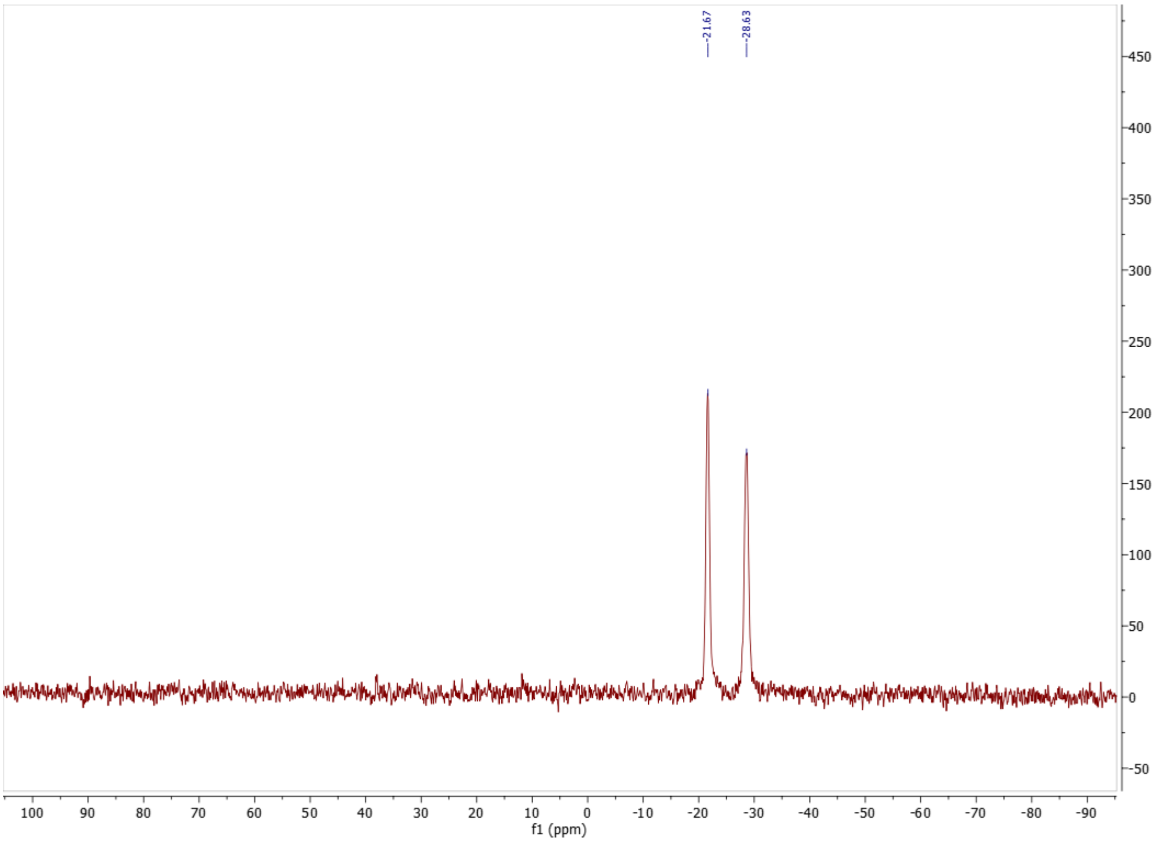

Supplementary Figure 2: ^{9}Be NMR (70 MHz) spectrum of compound **2** in C_6D_6 at 298 K.

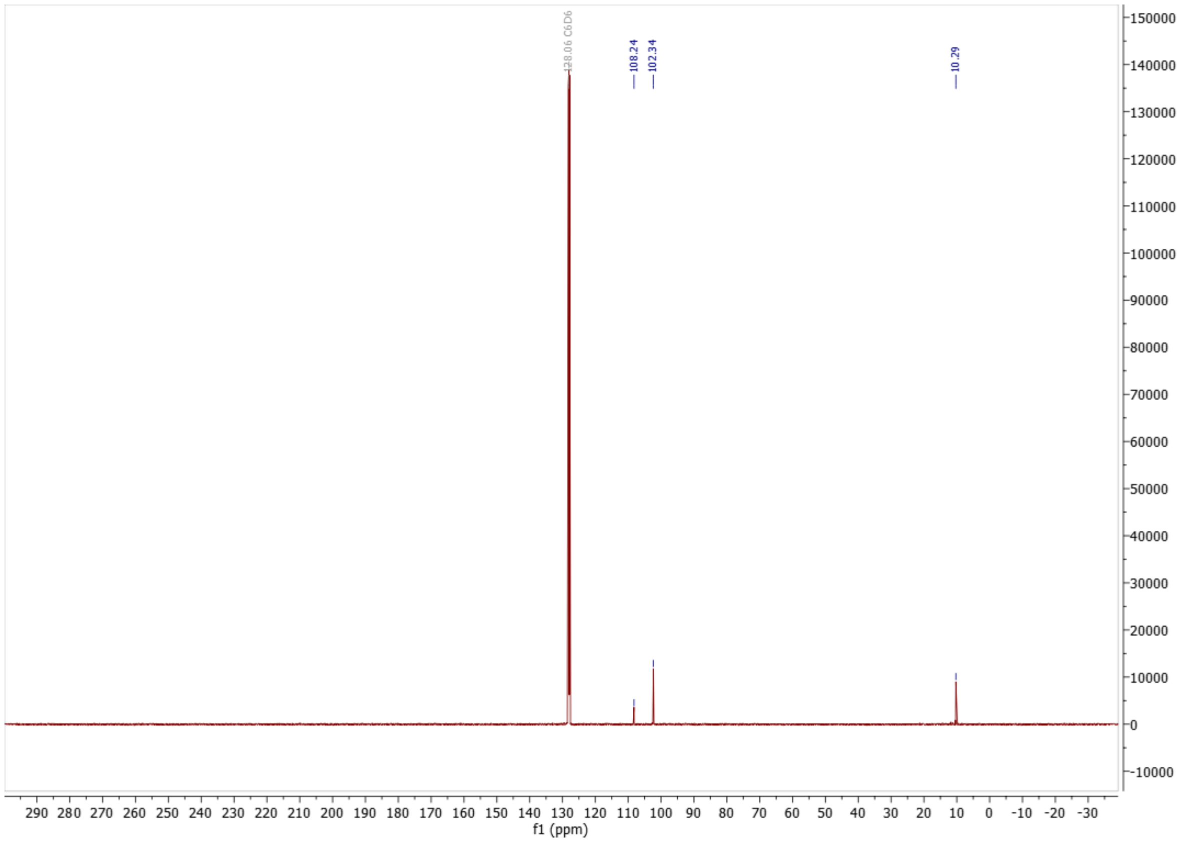

Supplementary Figure 3: ^{13}C {^{1}H} NMR (101 MHz) spectrum of compound **2** in C_6D_6 at 298 K.

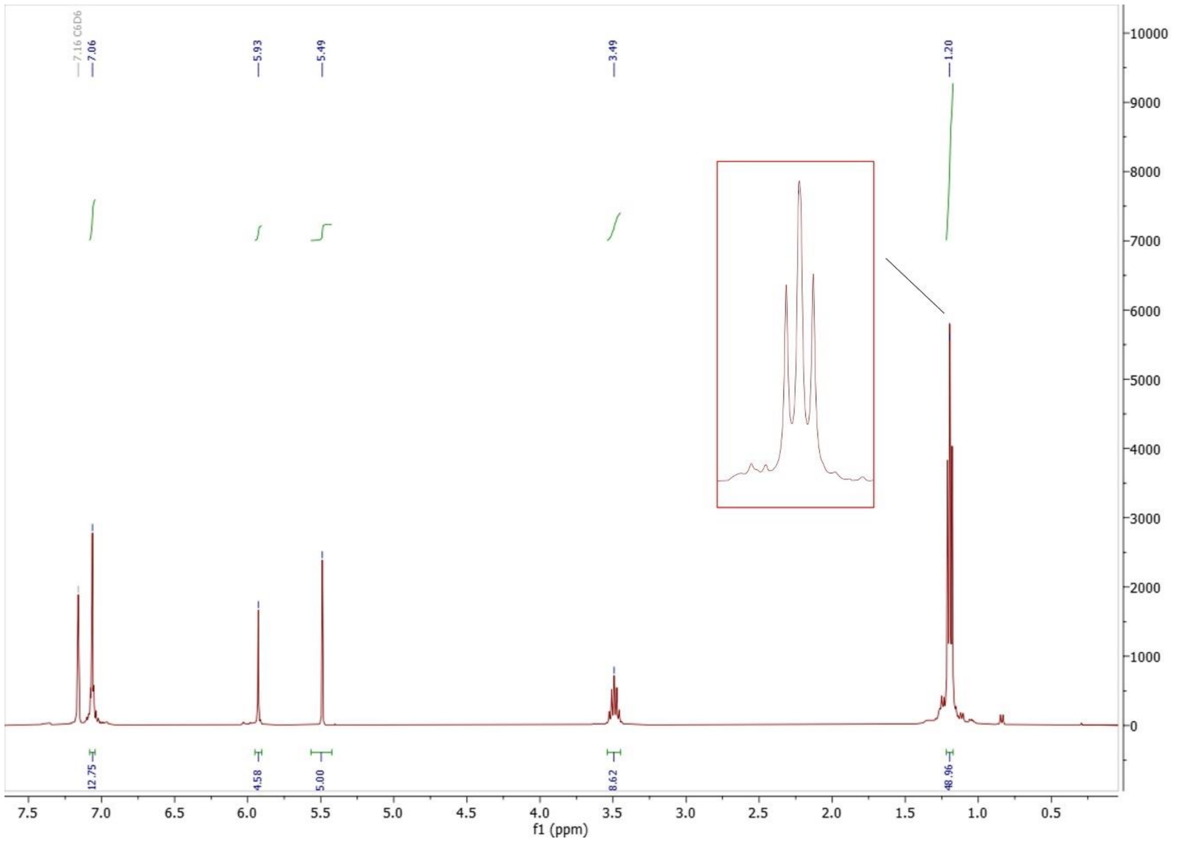

Supplementary Figure 4: ^{1}H NMR (400 MHz) spectrum of compound **3** in C_6D_6 at 298 K. The region from 1.15 – 1.25 ppm is also shown in an expanded view.

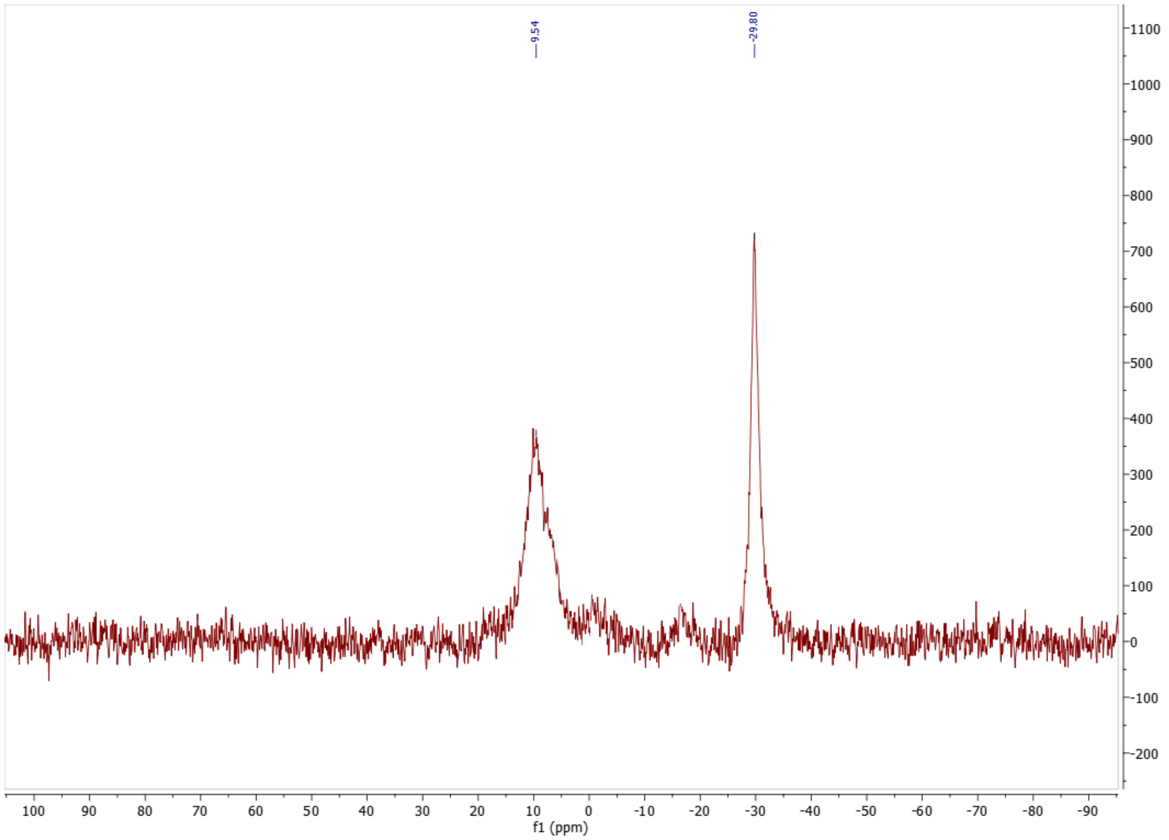

Supplementary Figure 5: ^{9}Be NMR (70 MHz) spectrum of compound **3** in C_6D_6 at 298 K.

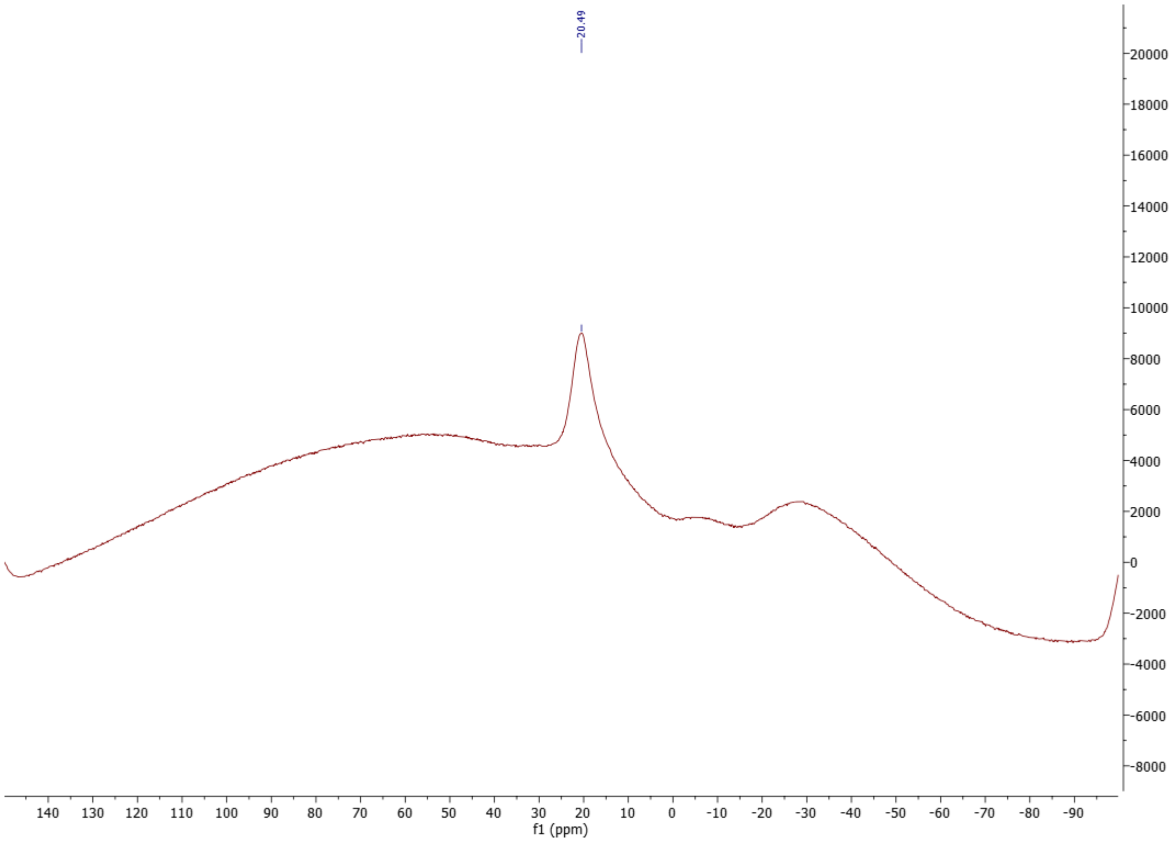

Supplementary Figure 6: ^{11}B NMR (128 MHz) spectrum of compound **3** in C_6D_6 at 298 K.

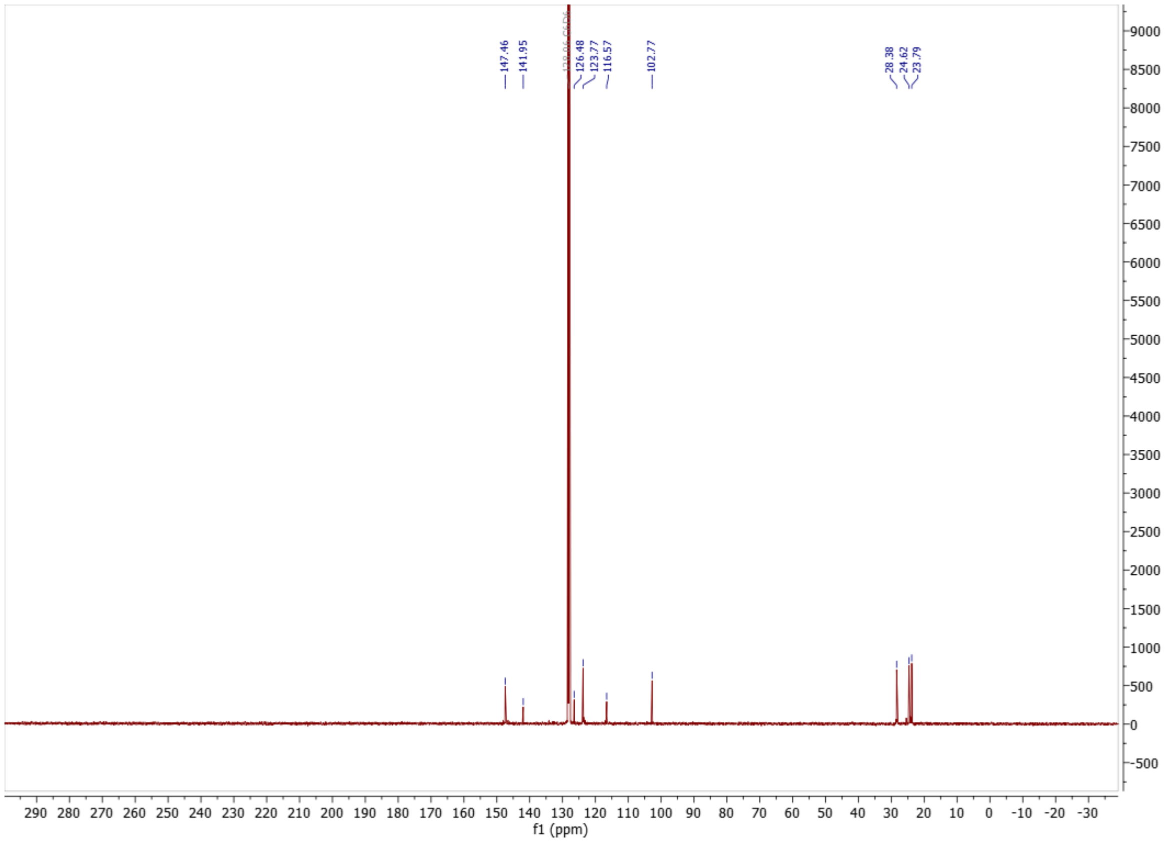

Supplementary Figure 7: $^{13}C\ \{^{1}H\}$ NMR (101 MHz) spectrum of compound **3** in C_6D_6 at 298 K.

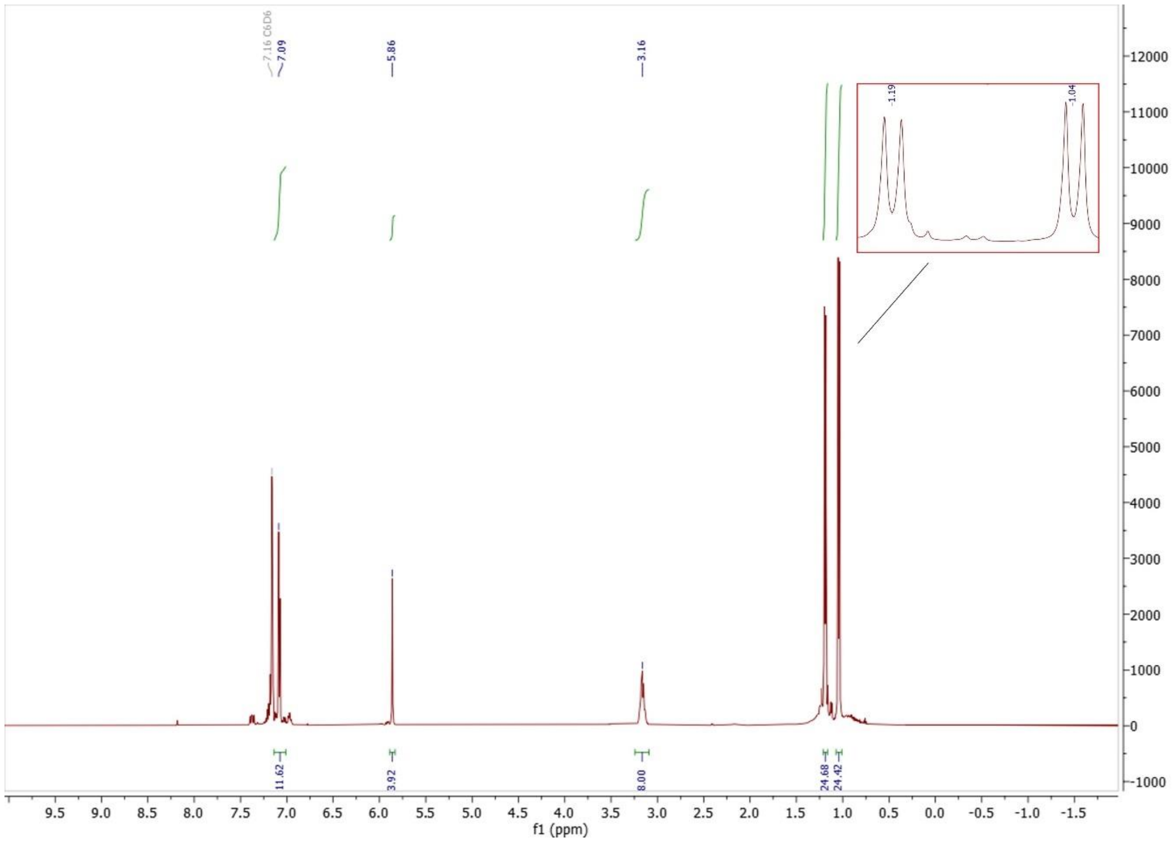

Supplementary Figure 8: ^{1}H NMR (400 MHz) spectrum of compound **4** in C_6D_6 at 298 K. The region from 1.00 – 1.25 ppm is also shown in an expanded view.

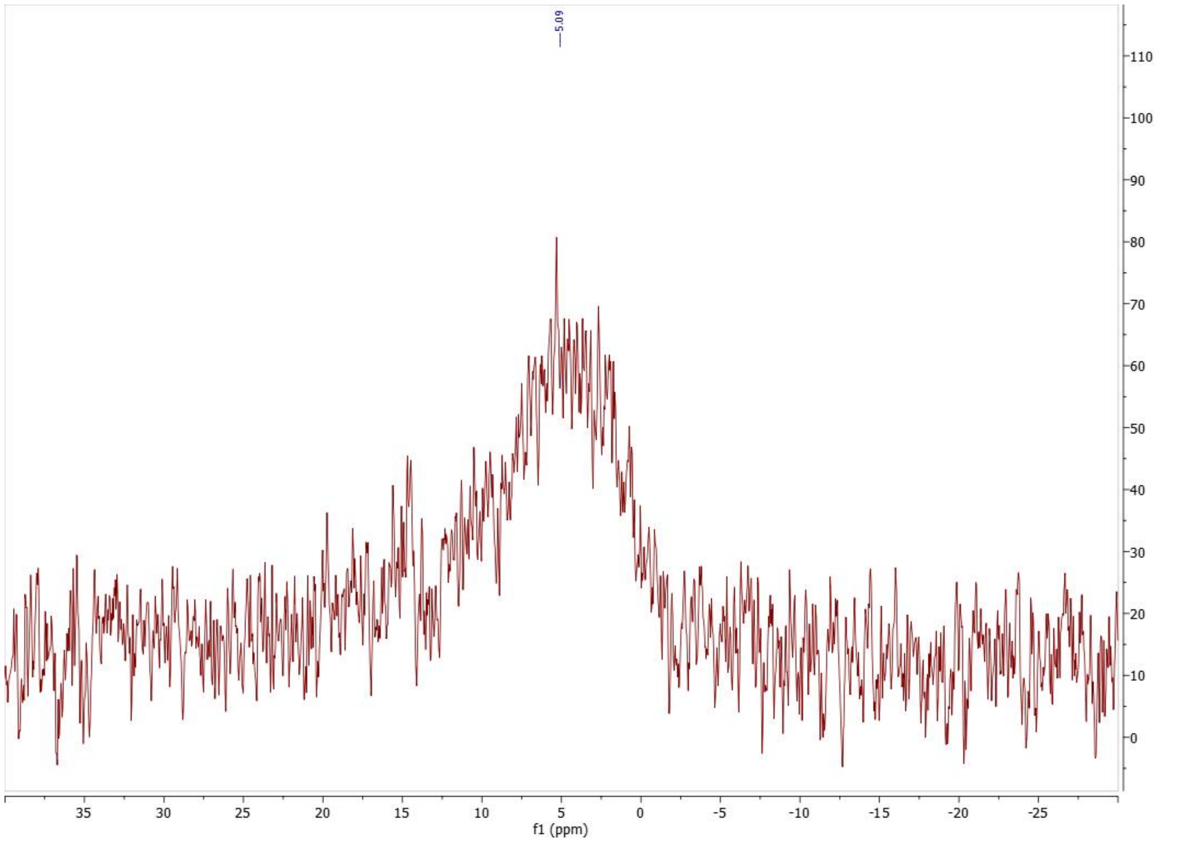

Supplementary Figure 9: ^{9}Be NMR (70 MHz) spectrum of compound **4** in C_6D_6 at 298 K.

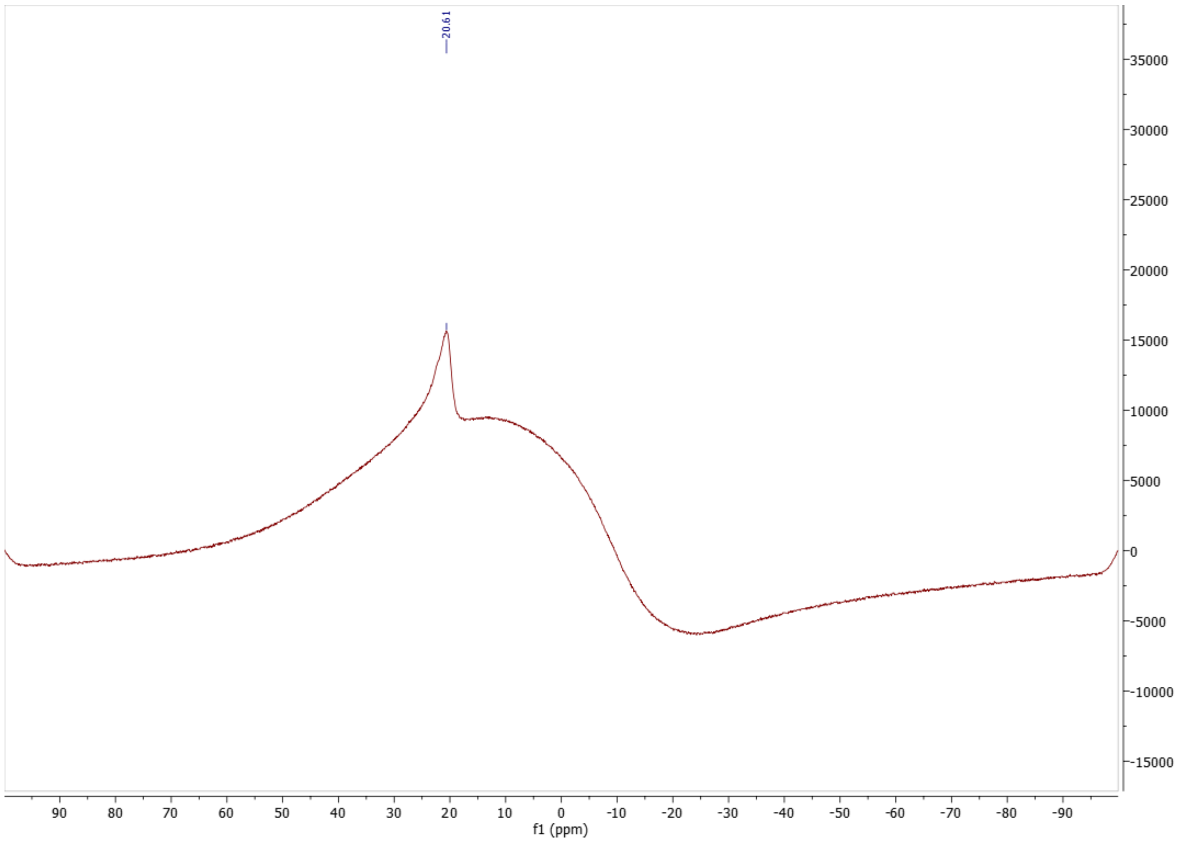

Supplementary Figure 10: ^{11}B NMR (128 MHz) spectrum of compound **4** in C_6D_6 at 298 K.

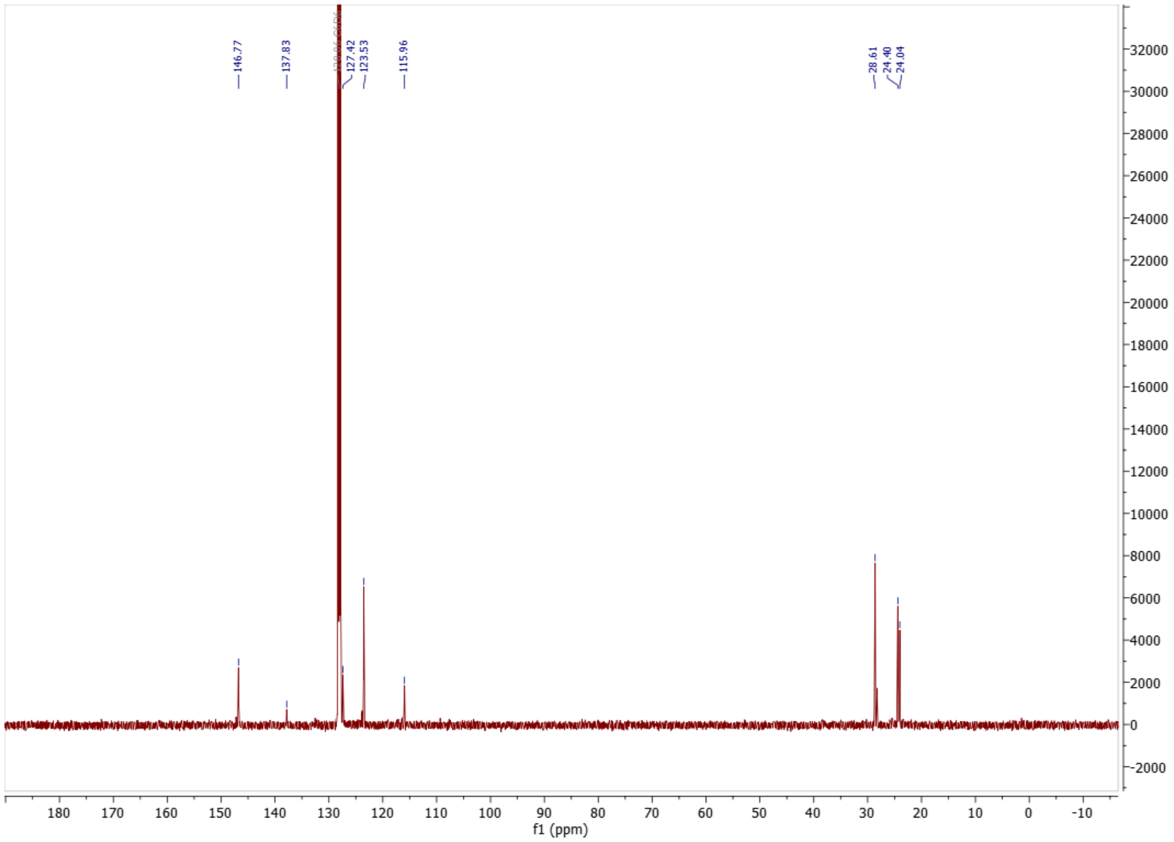

Supplementary Figure 11: ^{13}C {^{1}H} NMR (101 MHz) spectrum of compound **4** in C_6D_6 at 298 K.

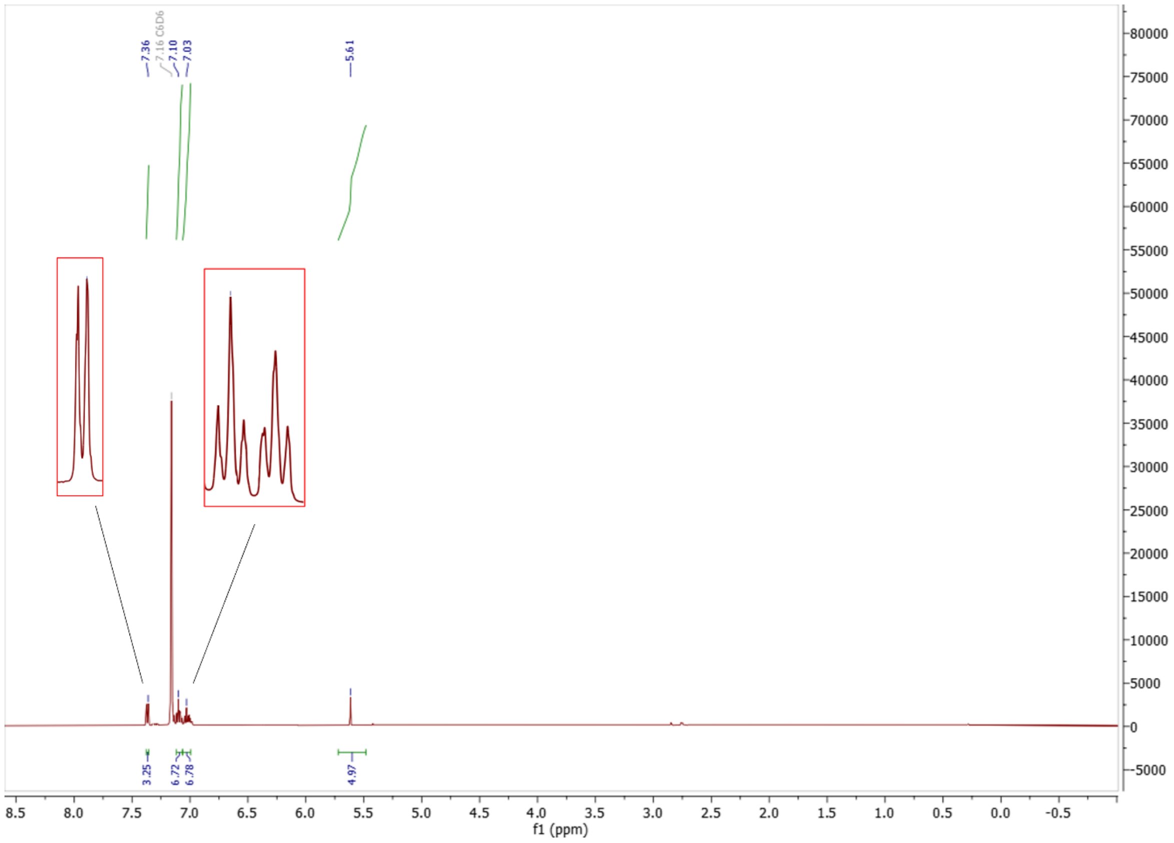

Supplementary Figure 12: ^{1}H NMR (400 MHz) spectrum of compound **5** in C_6D_6 at 298 K. The regions from 6.97 – 7.15 ppm and 7.35 – 7.40 ppm are also shown in an expanded view.

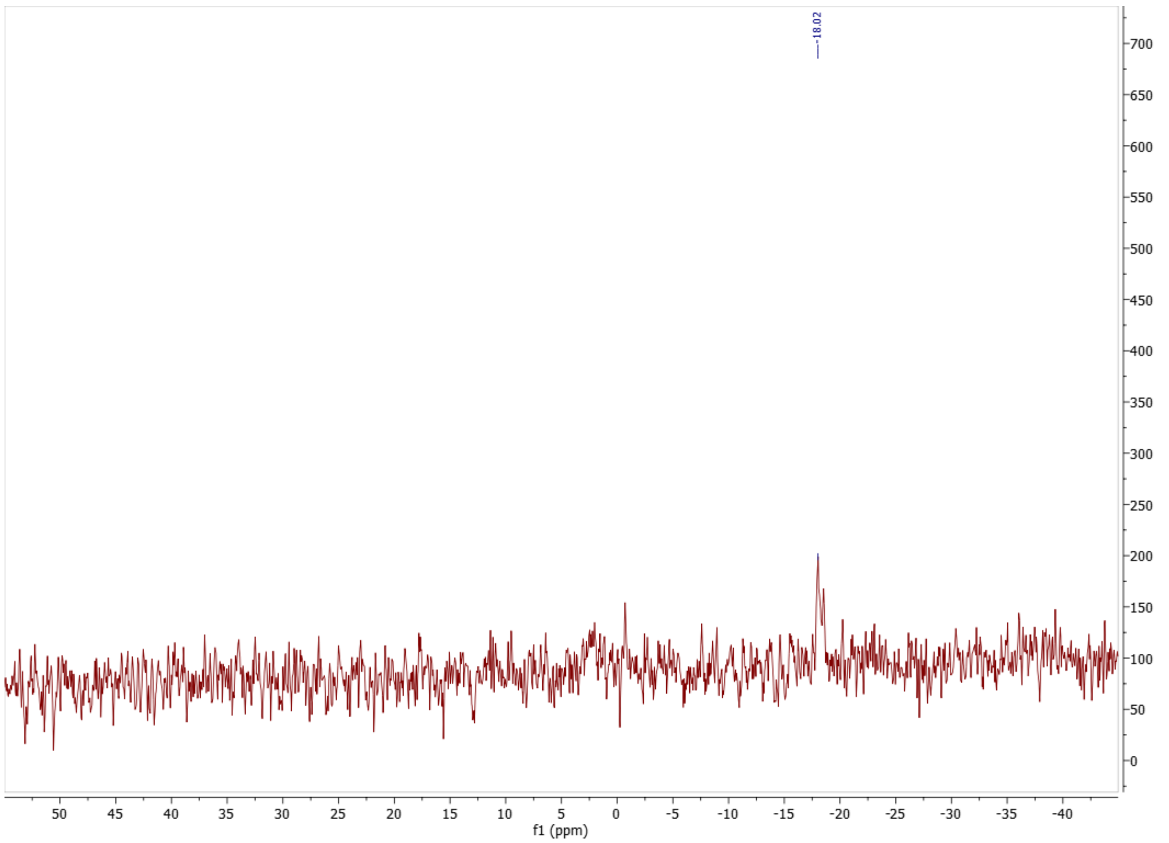

Supplementary Figure 13: ^{9}Be NMR (70 MHz) spectrum of compound **5** in C_6D_6 at 298 K.

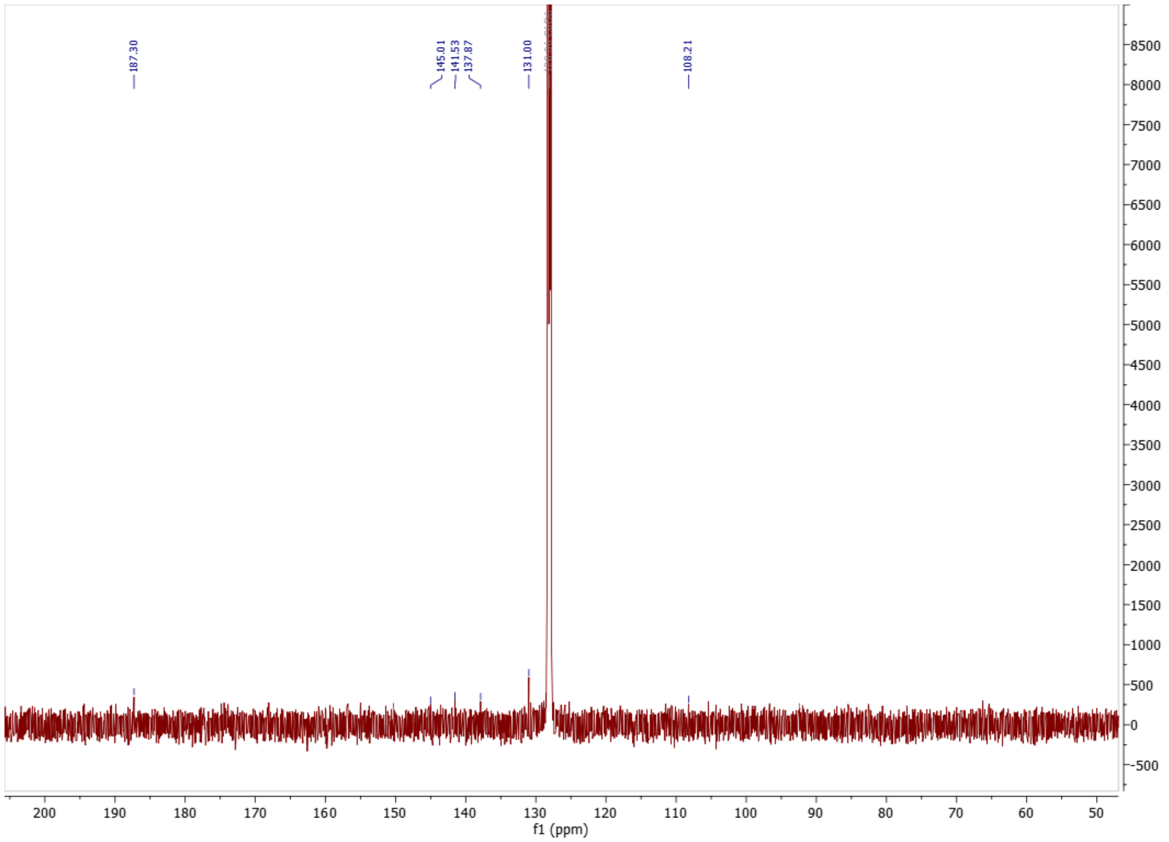

Supplementary Figure 14: ^{13}C {^{1}H} NMR (101 MHz) spectrum of compound **5** in C_6D_6 at 298 K.

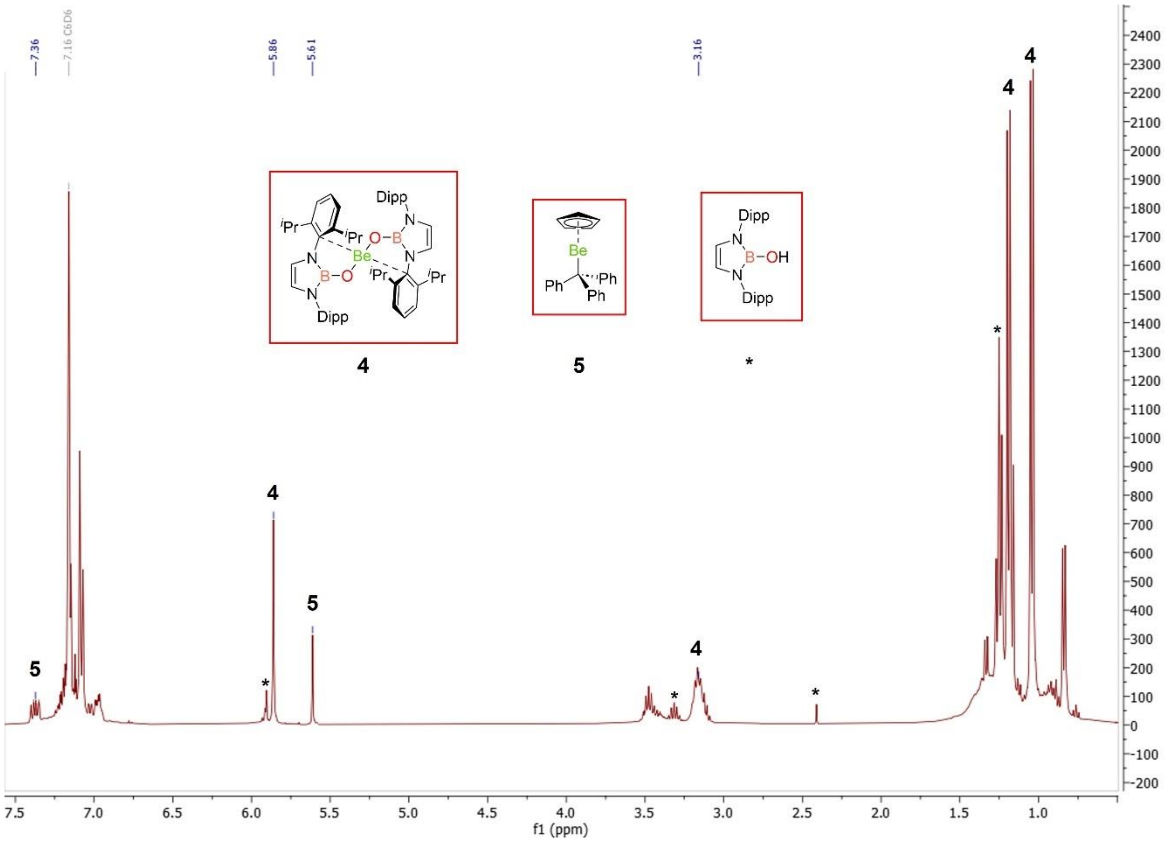

Supplementary Figure 15: Crude ^{1}H NMR (400 MHz) spectrum of the reaction between **3** and $[CPh_3][B(C_6F_5)_4]$ at 298 K.[1]

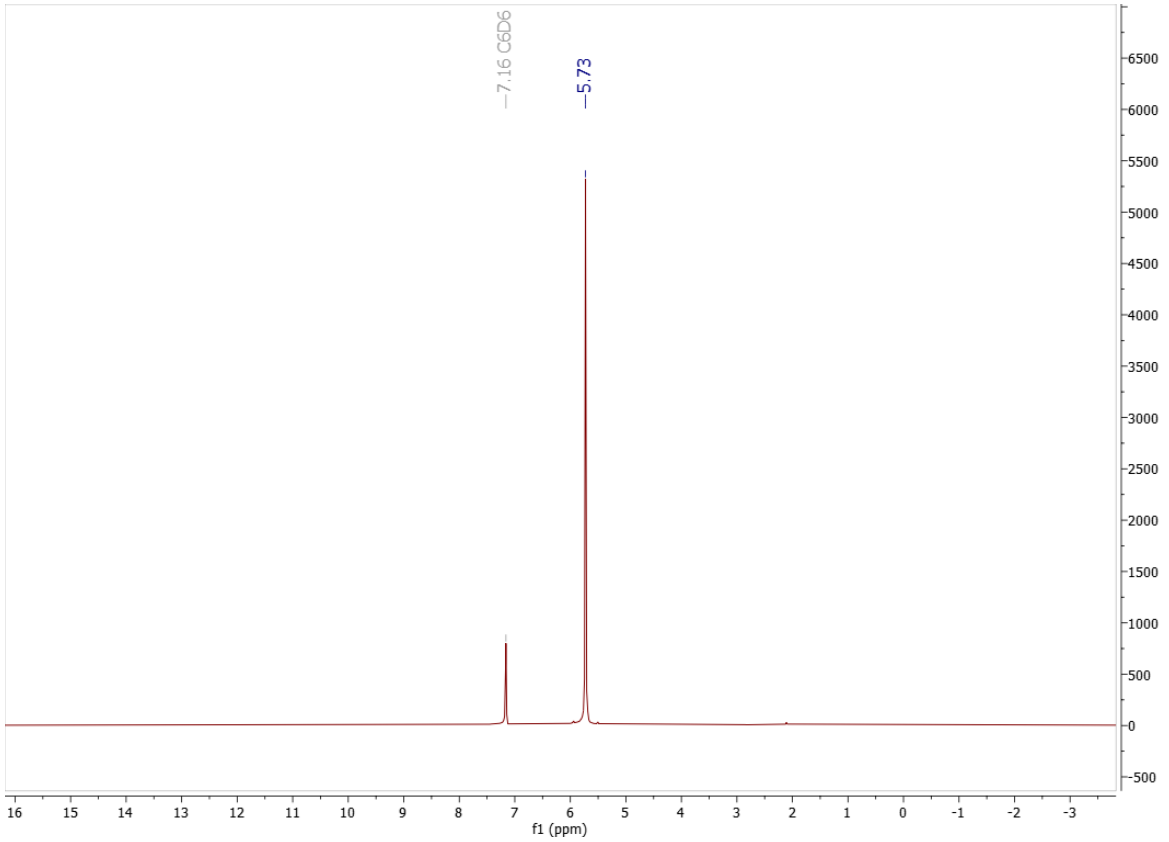

Supplementary Figure 16: ^{1}H NMR (400 MHz) spectrum of compound **1** in C_6D_6 at 298 K.

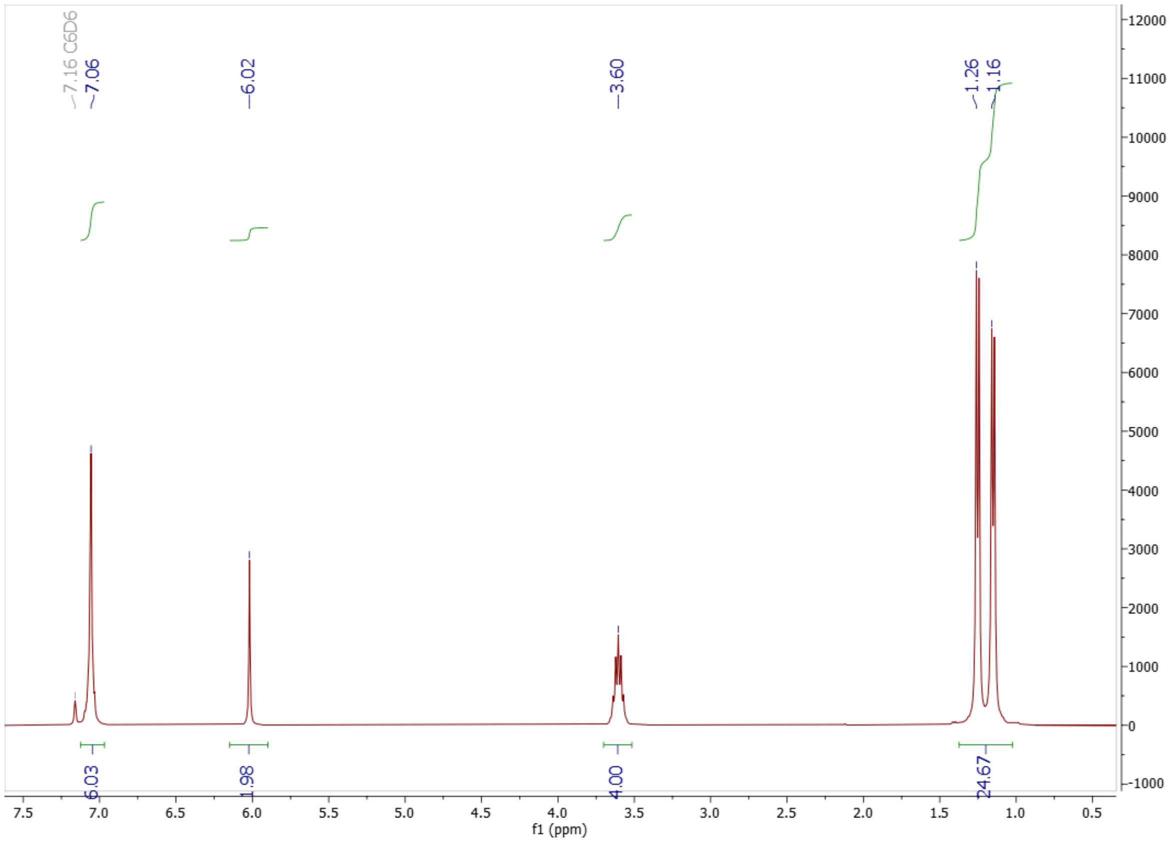

Supplementary Figure 17: ^{1}H NMR (400 MHz) spectrum of K-NHBO in C_6D_6 at 298 K.

Crystallographic Data

Supplementary Table 1: Selected X-ray data collection and refinement parameters

	**2**	**3**	**4**	**5**
Formula	C15 H20 Be2	C57 H77 B2 Be2 K N4 O2	C52 H72 B2 Be N4 O2	C24 H20 Be
Fw (g mol^{-1})	218.33	928.96	815.76	317.41
Cell setting	triclinic	monoclinic	orthorhombic	trigonal
Space group	$P\bar{1}$	$C2/c$	$Pccn$	$R\bar{3}$
a (Å)	8.6287(4)	24.6183(4)	15.63010(10)	11.23020(10)
b (Å)	12.8408(6)	12.9582(2)	18.58010(10)	11.23020(10)
c (Å)	13.2334(5)	18.3627(3)	16.80210(10)	23.9227(2)
α (°)	98.098(4)	90	90	90
β (°)	97.174(3)	110.923(2)	90	90
γ (°)	96.751(4)	90	90	120
V (Å^{-3})	1426.57(11)	5471.60(17)	4879.48(5)	2612.86(5)
Z	4	4	4	6
ρ_{calc} (g cm^{-3})	1.017	1.128	1.110	1.210
Radiation, λ (Å)	1.54184	1.54184	1.54184	1.54184
μ (mm^{-1})	0.391	1.169	0.502	0.502
$R_{(int)}$	0.0515	0.0391	0.0360	0.0248
Parameters	317	494	306	107
R_1 (all data/I >2σ(I))	0.0741/0.0496	0.0433/0.0359	0.0411/0.0393	0.0361/0.0348
ωR_2 (all data/I >2σ(I))	0.1463/0.1308	0.1008/0.0958	0.1011/0.0998	0.0876/0.0863
GooF	1.034	1.0288	1.051	1.076
T (K)	150.0(2)	100.0(2)	100.0(2)	100.0(2)
CCDC Deposition No.	2324716	2324715	2324717	2324714

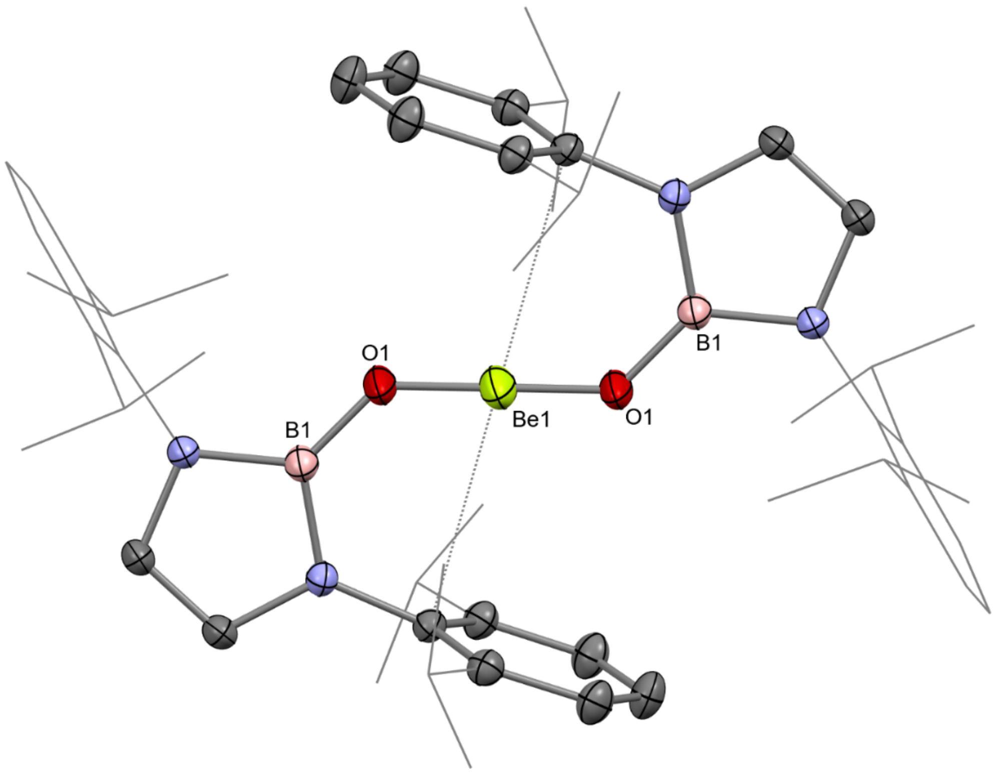

Supplementary Figure 18: Molecular structure of **4** in the solid state as determined by X-ray crystallography. Thermal ellipsoids set at 50% probability; hydrogen atoms omitted and selected substituents shown in wireframe format for clarity.

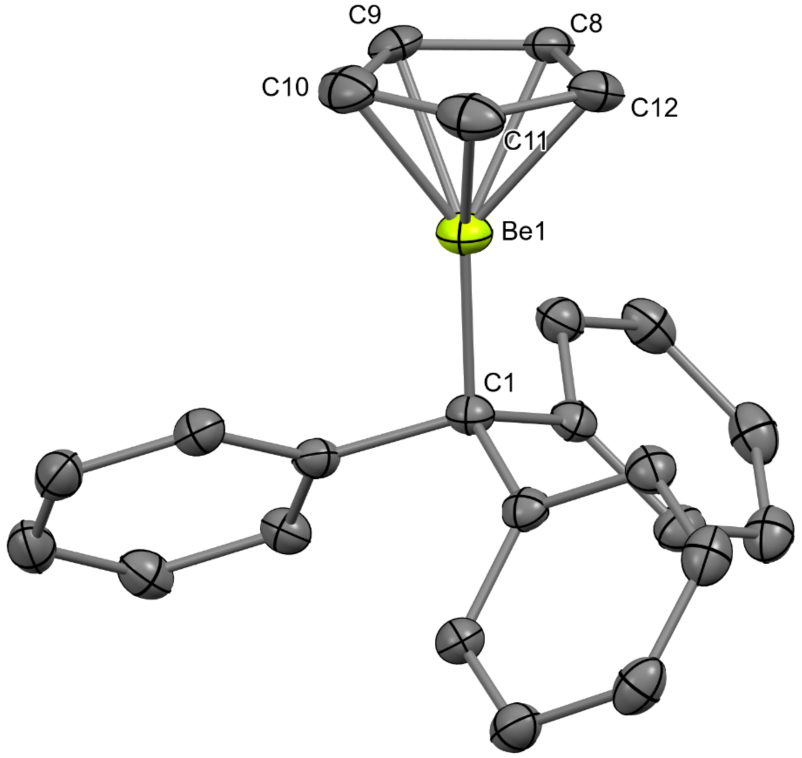

Supplementary Figure 19: Molecular structure of **5** in the solid state as determined by X-ray crystallography. Thermal ellipsoids set at 50% probability; hydrogen atoms omitted for clarity.

^{9}Be NMR Chemical Shifts – CpBeR Complexes

^{9}Be NMR chemical shift is very sensitive to the degree of electron density at a particular beryllium centre.[2,3] Of relevance here is the ^{9}Be NMR chemical shift of a cyclopentadienyl-coordinated beryllium centre. Increasingly upfield ^{9}Be NMR signals correspond to beryllium centres which are more electron-rich/reduced.[4] Indeed, the data for a series a series of (otherwise geometrically identical) compounds of the type CpBeX are presented in Supplementary Table 2. It could be proposed that the assignment of formal oxidation state gradually changes from Be(II) to Be(I) on transitioning from electronegative X-groups (e.g. X = Cl), to electropositive X-groups (e.g. X = aluminyl), with the unambiguous extension of this being X = CpBe (i.e., the *bona fide* Be(I) compound **1**). Indeed, the NMR data illustrates the step-wise formal reduction of beryllium, from the less powerful sigma donors (chloride/methyl), to intermediate (silyl- or gallyl-ligands), to aluminyl complex, and to **3**, in which Be_{Cp} can be considered to be in the 0-oxidation state.

Supplementary Table 2: ^{9}Be NMR chemical shifts (in C_6D_6) for a range of CpBeR species and the Pauling electronegativity of the atom from each group which is bonded to beryllium.[2]

**R-Group (CpBeR)**	**δ_{9Be} / ppm**	**Pauling Electroneg. of Donor Atom**
[K(NHBO)$_2$]Be (**3**)	–29.8	1.57
[(NON)Al]	–28.8	1.61
Cp*Be (**2**)	–28.6	1.57
(^{Dipp}Nacnac)Zn	–27.7	1.65
Me_3Si	–27.7	1.90
CpBe (**1**)	–27.6	1.57
[$Me_2(SiMe_3)Si$]	–27.2	1.90
[(NON)Ga]	–26.9	1.81
[{NON}Al{(N^{i}Pr)$_2$C}]	–24.6	2.55
Me	–20.5	2.55
Cl	–19.5	3.16
Br	–19.5	2.96
Ph_3C (**5**)	–18.0	2.55

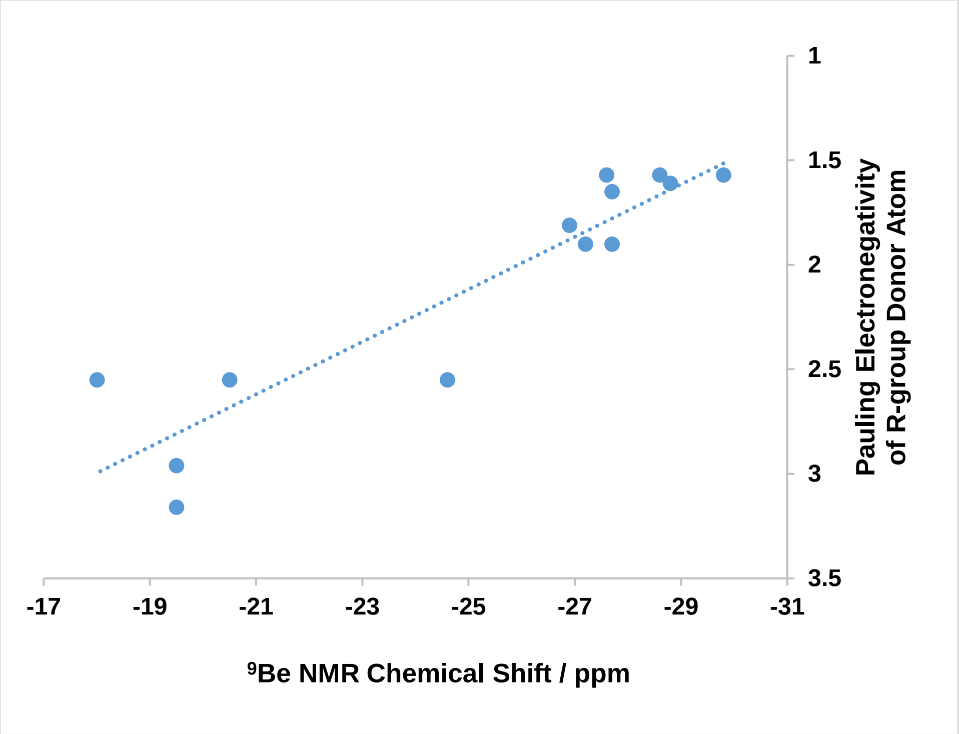

Supplementary Figure 20: Plot of ^{9}Be NMR chemical shifts for a range of CpBeR species *vs* the Pauling electronegativity of the atom from each group which is bonded to beryllium.

Computational Details

Relevant Molecular Orbitals

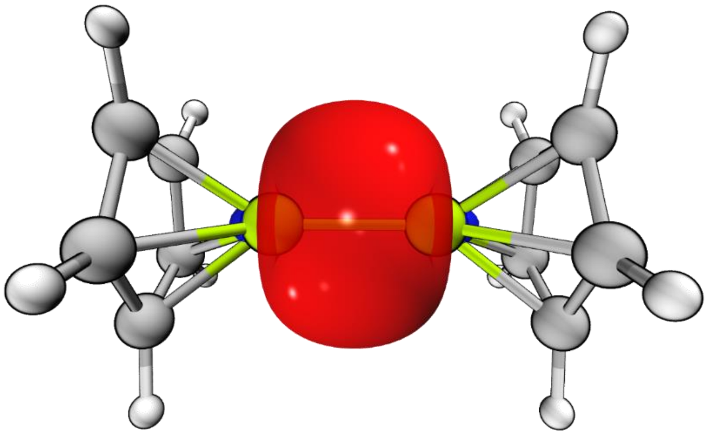

Supplementary Figure 21: HOMO of compound **1** (0.05 a.u.).

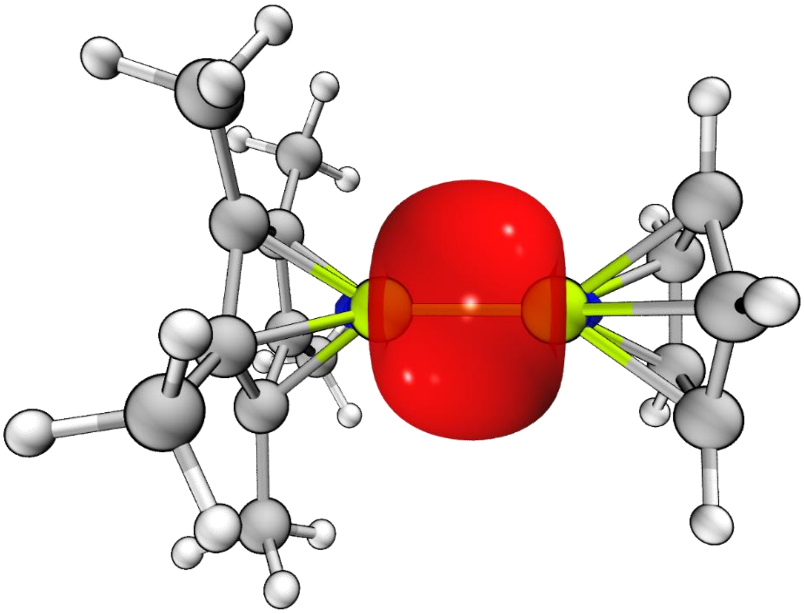

Supplementary Figure 22: HOMO of compound **2** (0.05 a.u.).

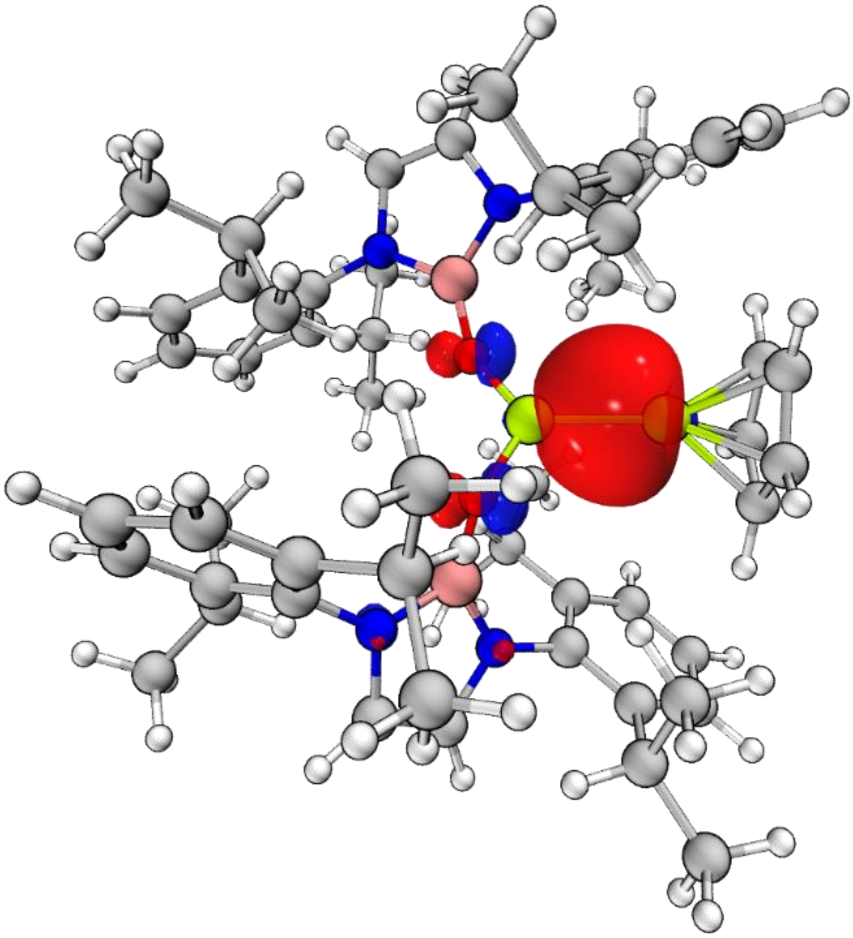

Supplementary Figure 23: HOMO of anion **3’** (0.05 a.u.).

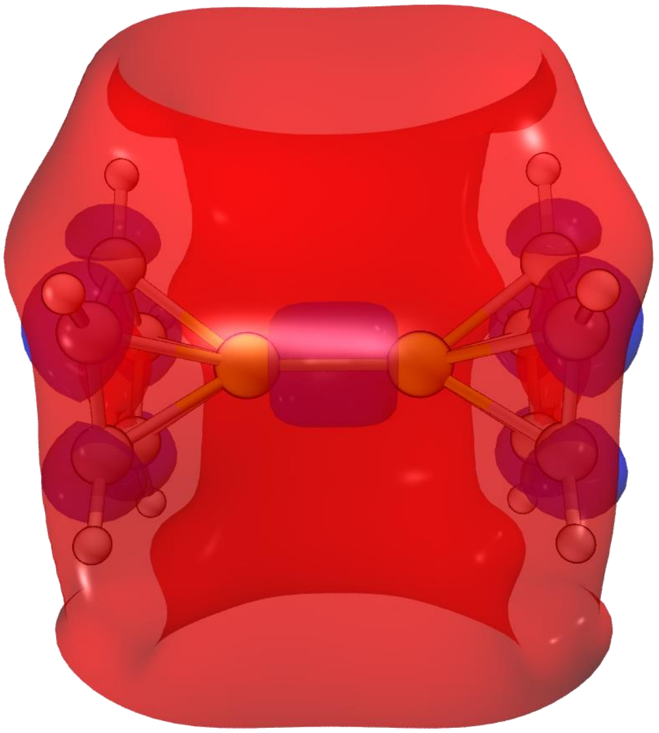

Supplementary Figure 24: LUMO of compound **1** (0.05 a.u.).

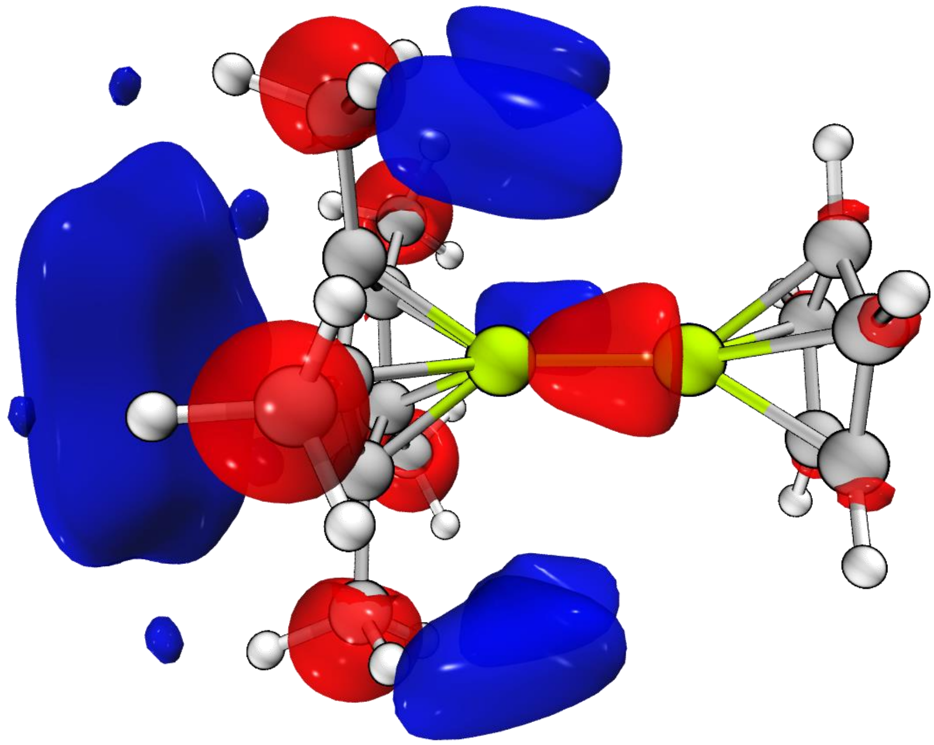

Supplementary Figure 25: LUMO of compound **2** (0.05 a.u.).

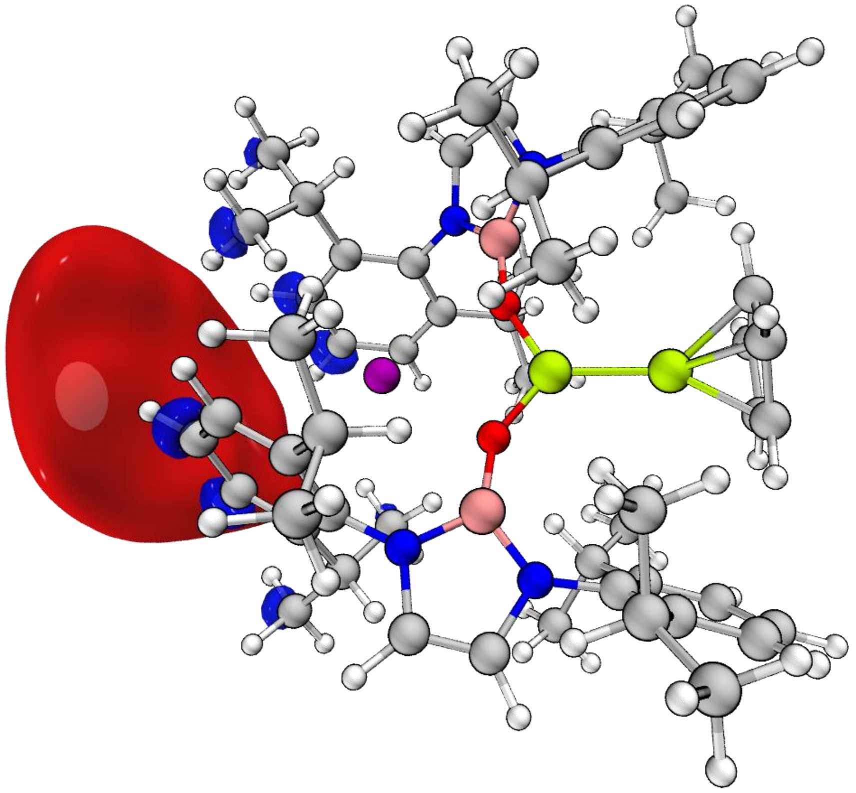

Supplementary Figure 26: LUMO of compound **3** (0.05 a.u.).

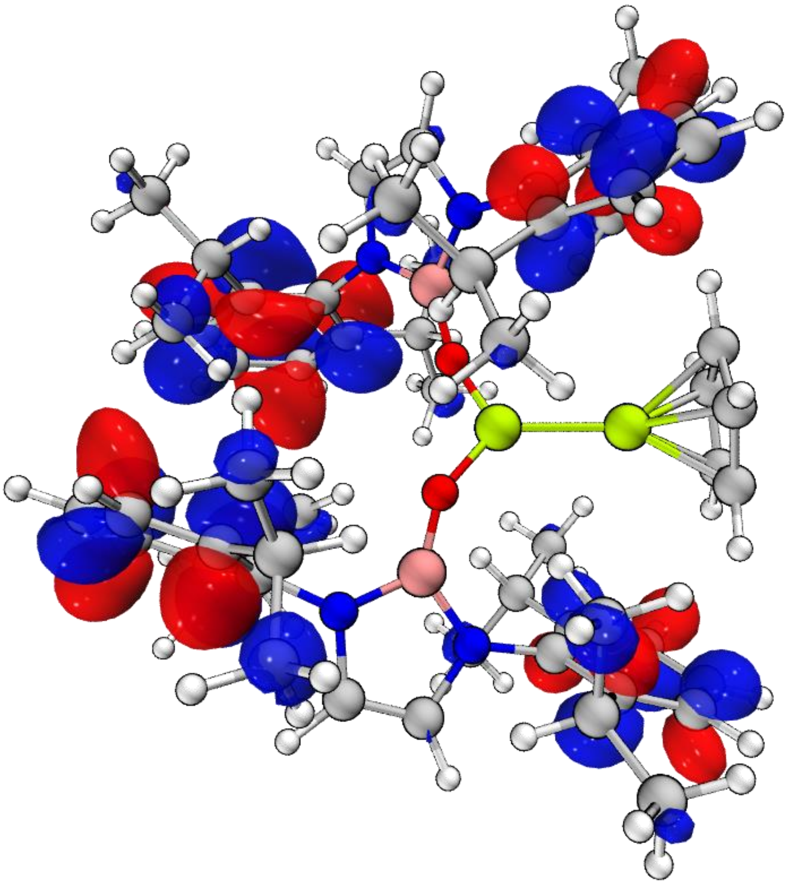

Supplementary Figure 27: LUMO of anion **3’** (0.05 a.u.).

Quantum Theory of Atoms in Molecules Calculations

Supplementary Table 3: Summary of QTAIM data generated for **1** – **3** and **3’**. All values in a.u..

	**1**		**2**			**3**	**3’**
**Critical Point**	**Be$_{Cp}$-NNA**	**NNA**	**Be$_{Cp}$-NNA**	**Be$_{Cp*}$-NNA**	**NNA**	**Be-Be**	**Be-Be**
**ρ_{bcp} / e$^-$ Bohr^{-3}**	0.067	0.068	0.067	0.066	0.067	0.060	0.057
**KE (L)**	0.020	0.002	0.018	0.021	0.003	0.019	0.020
**KE (H)**	0.039	0.031	0.039	0.037	0.033	0.034	0.031
**V**	-0.059	-0.033	-0.057	-0.059	-0.035	-0.052	-0.052
**E**	-0.039	-0.031	-0.039	-0.037	-0.033	-0.034	-0.031
**$\nabla^2\rho_{bcp}$ / e$^-$ Bohr^{-5}**	-0.076	-0.116	-0.085	-0.064	-0.119	-0.059	-0.044
**ELF**	0.716	0.996	0.758	0.671	0.992	0.663	0.596
**LOL**	0.614	0.942	0.639	0.588	0.917	0.584	0.548

Supplementary Table 4: Charge distribution data (Bader) and contributions to disynaptic basin corresponding to Be-Be bond (ELF) for compounds **1** and **2**.

**Compound 1**			**Compound 2**		
	**Bader Charge**	**Contribution to Disynaptic Basin**		**Bader Charge**	**Contribution to Disynaptic Basin**
**NNA**	-1.17456	1.16844	**NNA**	-1.148042	1.14387
**Be**	1.420623	0.42623	**Be_{Cp}**	1.391892	0.45831
**Be**	1.42052	0.43131	**Be_{Cp^*}**	1.427303	0.40704

Supplementary Table 5: Charge distribution data (Bader) and contributions to disynaptic basin corresponding to Be-Be bond (ELF) for compounds **3** and **3'**.

**Compound 3**			**Compound 3'**		
	**Bader charge**	**Contribution to Disynaptic Basin**		**Bader Charge**	**Contribution to Disynaptic Basin**
**NNA**	N/A	N/A	**NNA**	N/A	N/A
**Be_{NHBO}**	1.622375	0.24004	**Be_{NHBO}**	1.645432	1.68654
**Be_{Cp}**	0.188163	1.66613	**Be_{Cp}**	0.175772	0.21141

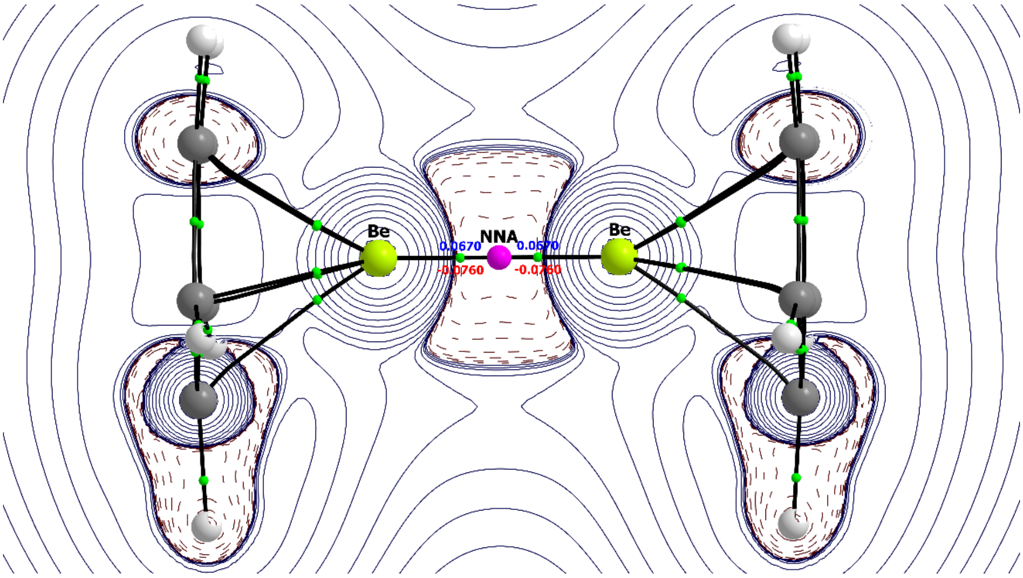

Supplementary Figure 28: Plot of QTAIM data generated for **1**. Bond paths (BPs) in black and bond critical points (BCPs) in green. Contour map of is of Laplacian ($\nabla^2\rho_{bcp}$). Values in red are $\nabla^2\rho_{bcp}$ (e^- $Bohr^{-5}$) and values in blue are ρ_{bcp} (electron density; e^- $Bohr^{-3}$) at the respective BCP. Ring critical points and prismatic critical points are omitted for clarity.

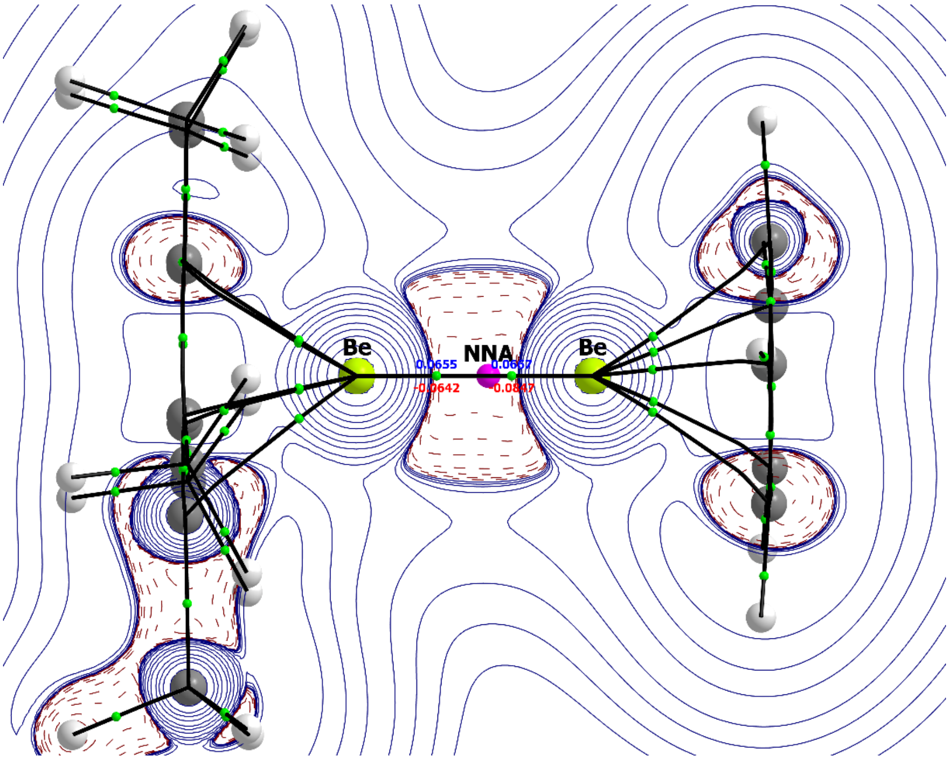

Supplementary Figure 29: Plot of QTAIM data generated for **2**. Bond paths (BPs) in black and bond critical points (BCPs) in green. Contour map of is of Laplacian ($\nabla^2\rho_{bcp}$). Values in red are $\nabla^2\rho_{bcp}$ (e^- $Bohr^{-5}$) and values in blue are ρ_{bcp} (electron density; e^- $Bohr^{-3}$) at the respective BCP. Ring critical points and prismatic critical points are omitted for clarity.

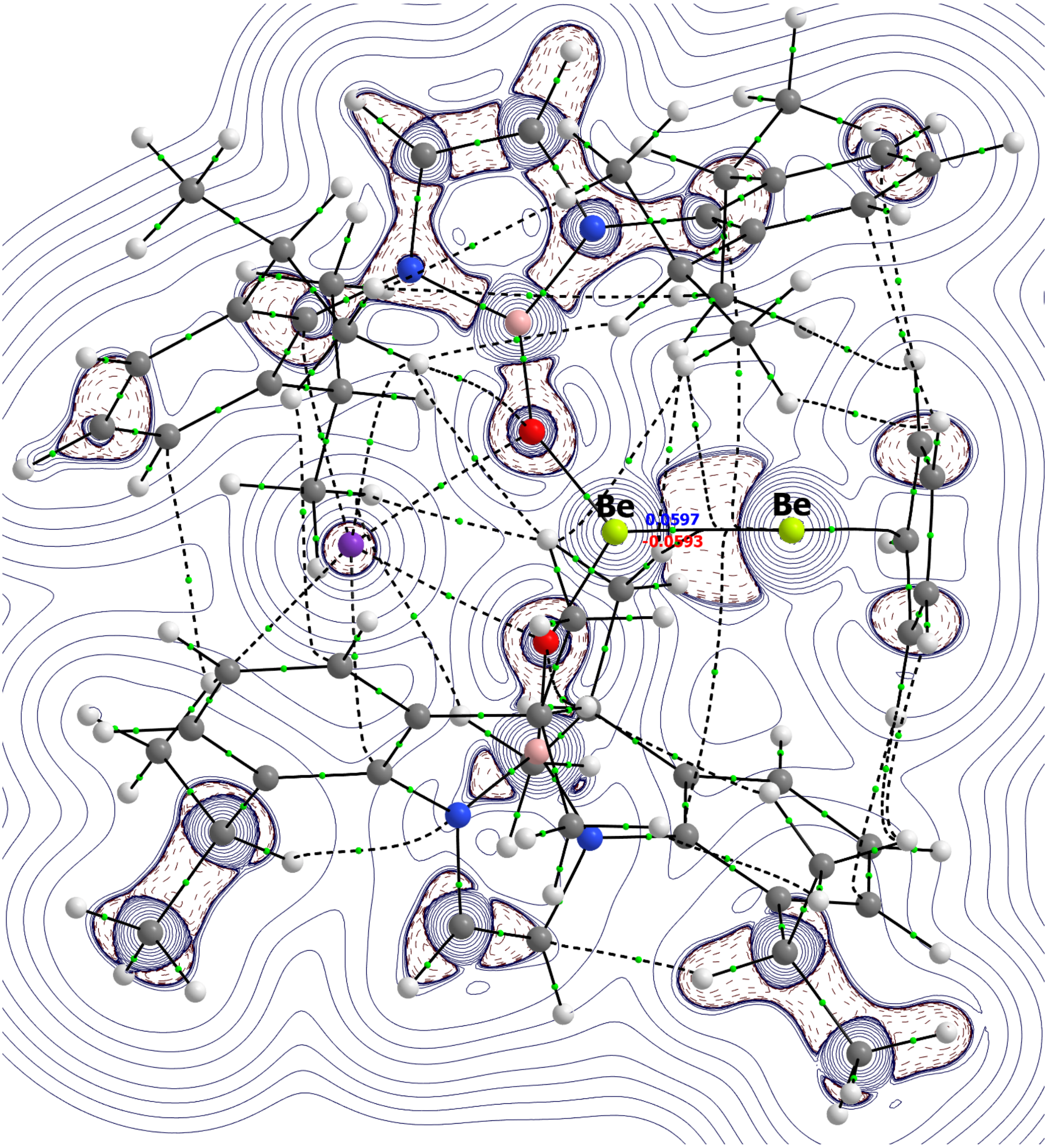

Supplementary Figure 30: Plot of QTAIM data generated for **3**. Bond paths (BPs) in black and bond critical points (BCPs) in green. Contour map of is of Laplacian ($\nabla^2\rho_{bcp}$). Values in red are $\nabla^2\rho_{bcp}$ (e^- $Bohr^{-5}$) and values in blue are ρ_{bcp} (electron density; e^- $Bohr^{-3}$) at the respective BCP. Ring critical points and prismatic critical points are omitted for clarity.

Electron Localisation Function Isosurfaces and Residual Electron Density Maps

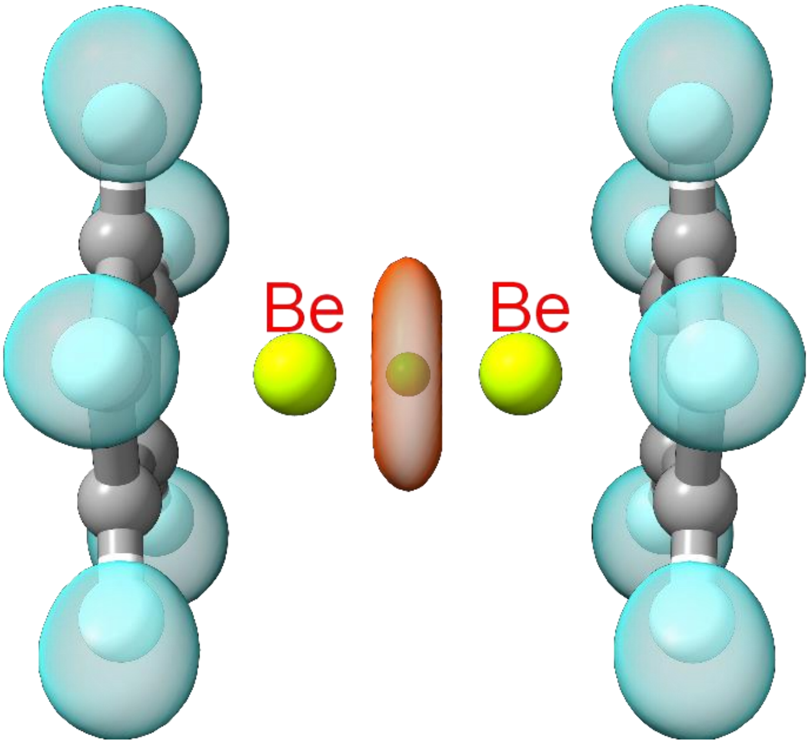

Supplementary Figure 31: ELF isosurface for **1** (0.7 a.u.). Blue represents the isosurfaces associated with the Cp ligands. Orange represents the isosurface associated with the Be–Be bonding basin.

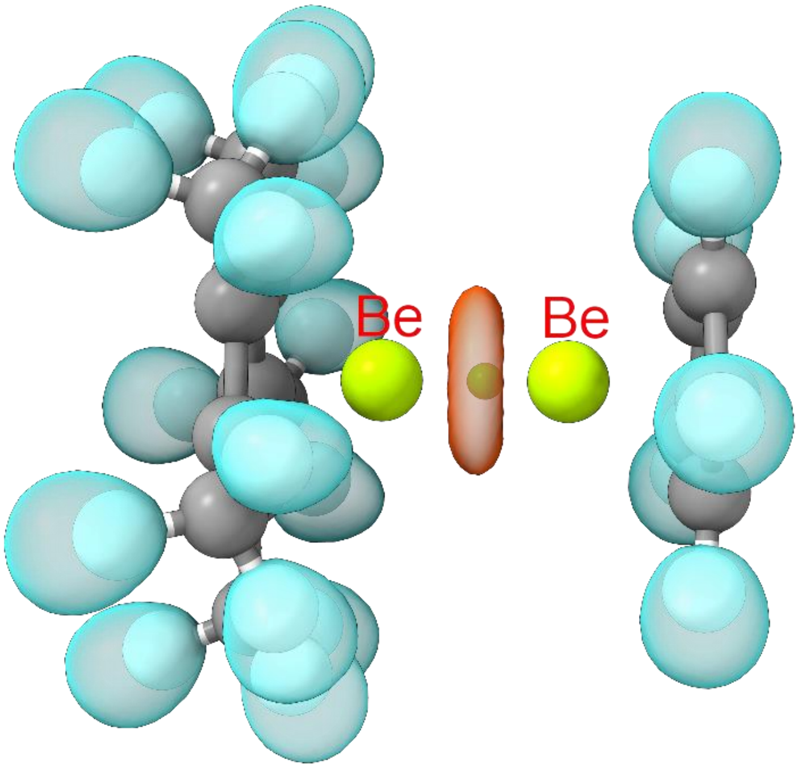

Supplementary Figure 32: ELF isosurface for **2** (0.7 a.u.). Blue represents the isosurfaces associated with the ligands. Orange represents the isosurface associated with the Be–Be bonding basin.

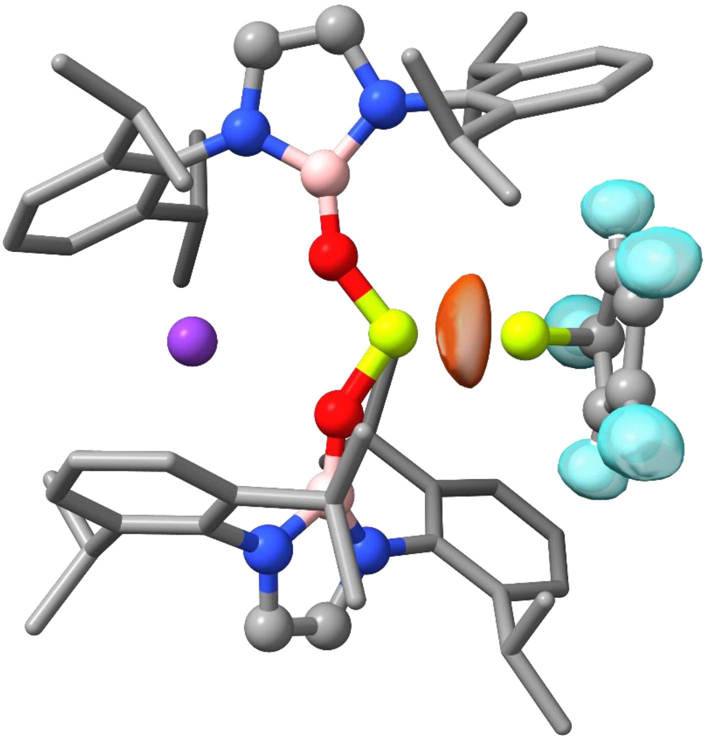

Supplementary Figure 33: ELF isosurface for **3** (0.7 a.u.). Blue represents the isosurfaces associated with the Cp ligand. Orange represents the isosurface associated with the Be–Be bonding basin.

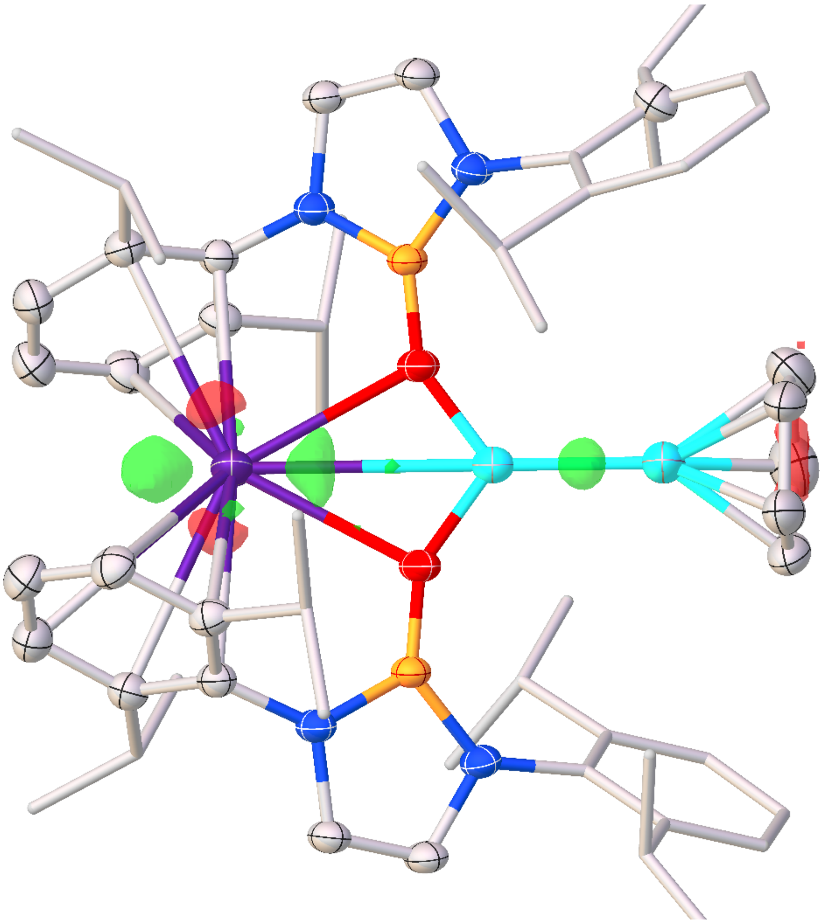

Supplementary Figure 34: Residual electron density map for **3** derived from SCXRD data. Threshold: ±0.31 e Å^{-3}

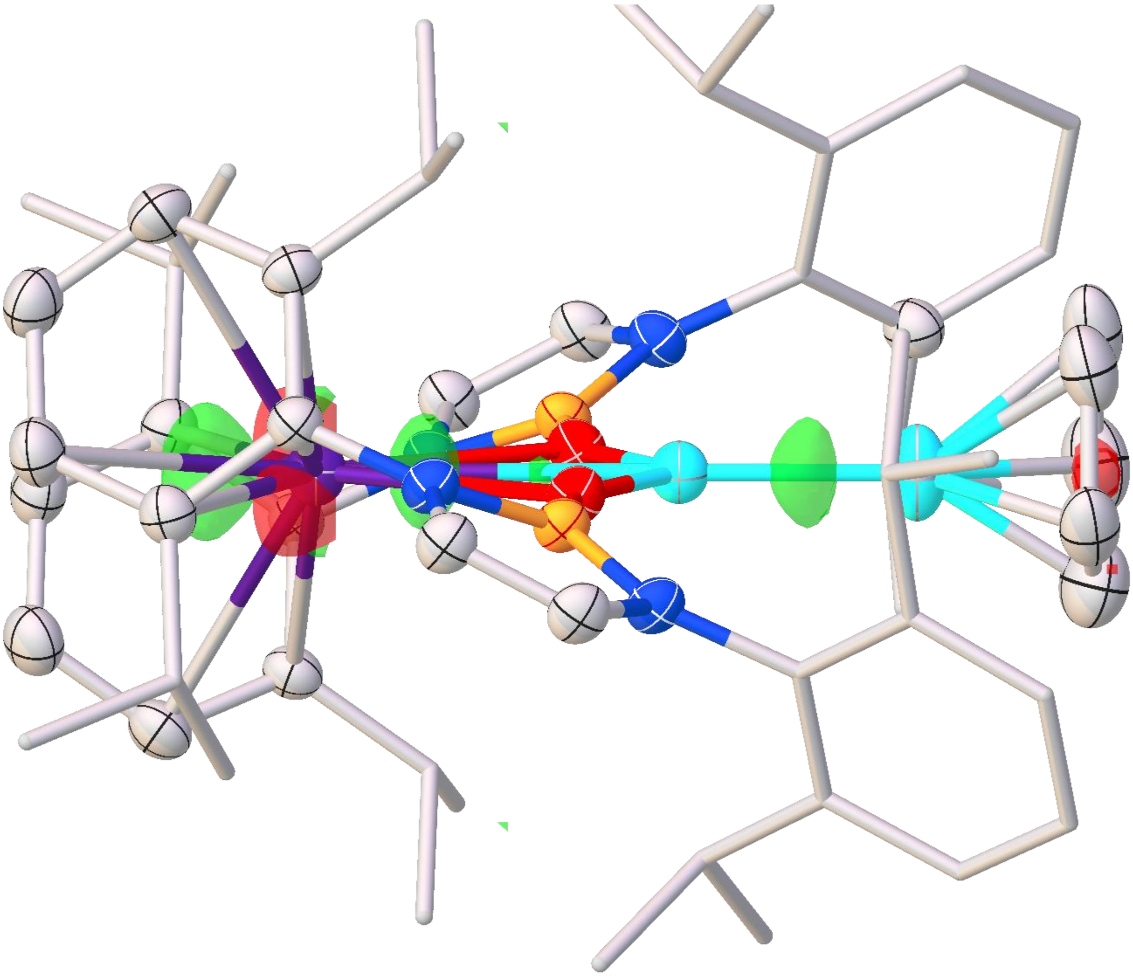

Supplementary Figure 35: Residual electron density map for **3** derived from SCXRD data. Threshold: ±0.31 e Å^{-3}

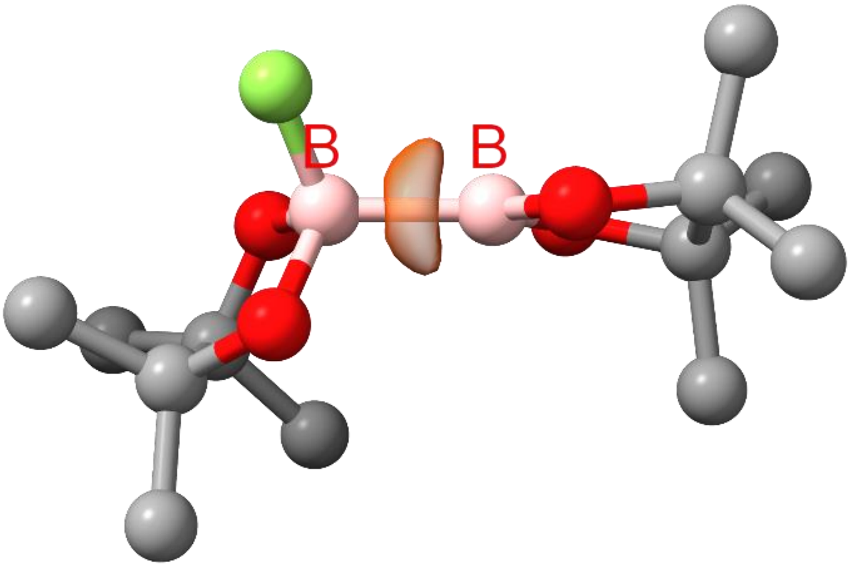

Supplementary Figure 36: ELF isosurface for **B** (0.7 a.u.). Orange represents the isosurface associated with the B–B bonding basin.

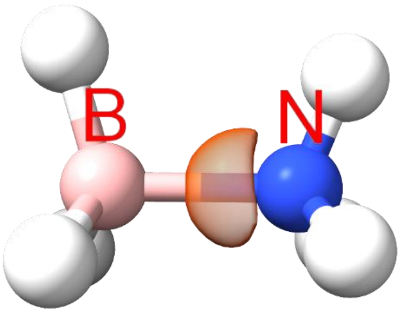

Supplementary Figure 37: ELF isosurface for **C** (0.7 a.u.). Orange represents the isosurface associated with the B–N bonding basin.

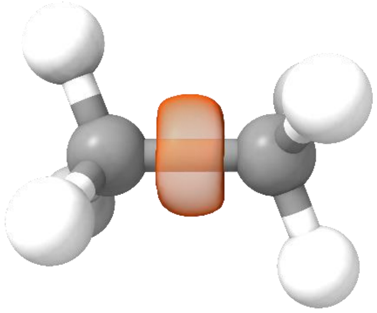

Supplementary Figure 38: ELF isosurface for $F_3C–CH_3$ (0.7 a.u.). Orange represents the isosurface associated with the C–C bonding basin.

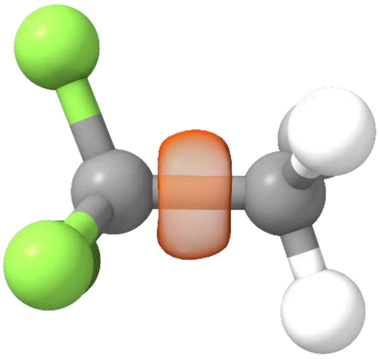

Supplementary Figure 39: ELF isosurface for $F_3C–CH_3$ (0.7 a.u.). Orange represents the isosurface associated with the C–C bonding basin.

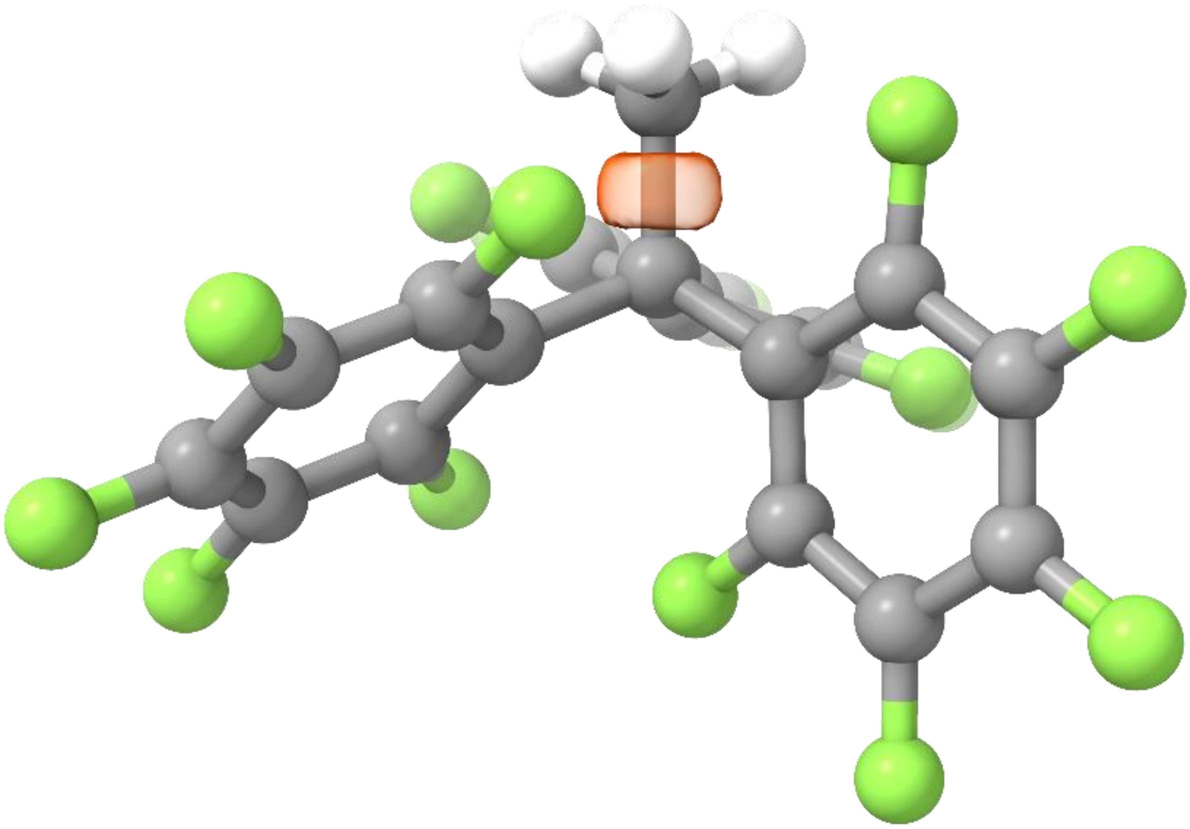

Supplementary Figure 40: ELF isosurface for $(C_6F_5)_3C–CH_3$ (0.7 a.u.). Orange represents the isosurface associated with the C–C bonding basin.

Natural Bond Orbital and Natural Population Analysis Calculations

Supplementary Table 6: Charge distribution (NPA) and Wiberg bond index (WBI) data for compounds **1** and **2**.

Compound 1		Compound 2	
	**NPA Charge**		**NPA Charge**
**Be$_{Cp}$**	0.85063	**Be$_{Cp}$**	0.85911
**Be$_{Cp}$**	0.85036	**Be$_{Cp*}$**	0.86709
**WBI (Be-Be)**	0.8933	**WBI (Be-Be)**	0.8962

Supplementary Table 7: Charge distribution (NPA) and Wiberg bond index (WBI) data for compounds **3** and **3'**.

Compound 3		Compound 3'	
	**NPA Charge**		**NPA Charge**
**Be$_{Cp}$**	1.15137	**Be$_{Cp}$**	1.17501
**Be$_{NHBO}$**	0.63978	**Be$_{NHBO}$**	0.60071
**WBI (Be-Be)**	0.8054	**WBI (Be-Be)**	0.7873

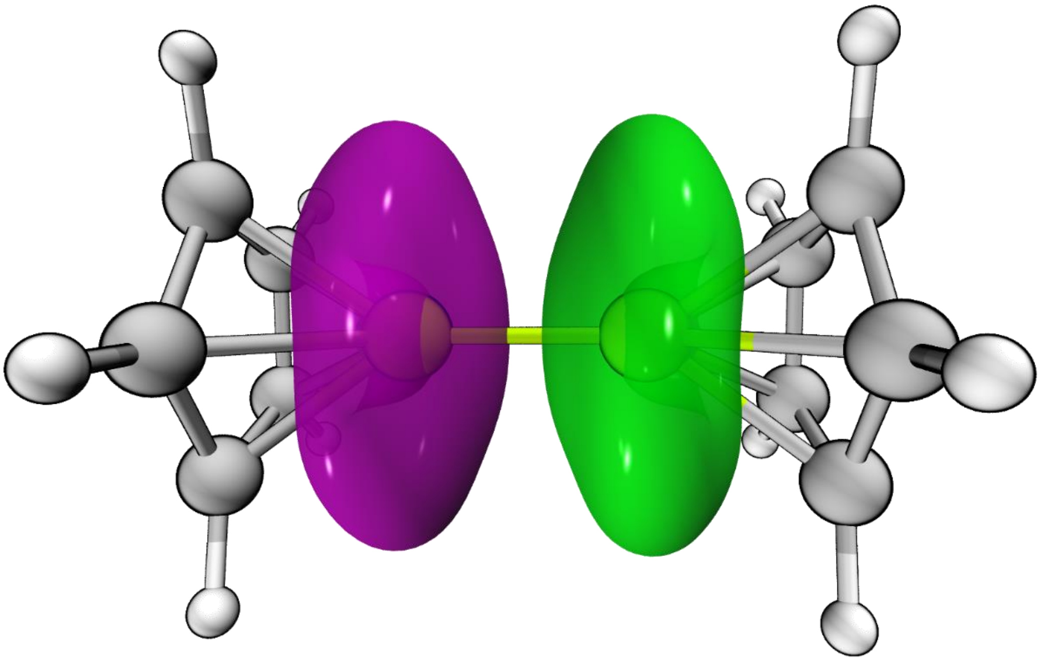

Supplementary Figure 41: NBO for the Be–Be interaction of **1** (0.05 a.u.). Contribution (Be$_{Cp}$:Be$_{Cp}$) to Be-Be bond: 50:50. Wiberg bond index: 0.89. Natural population analysis charges (Be$_{Cp}$, Be$_{Cp}$): 0.85, 0.85.

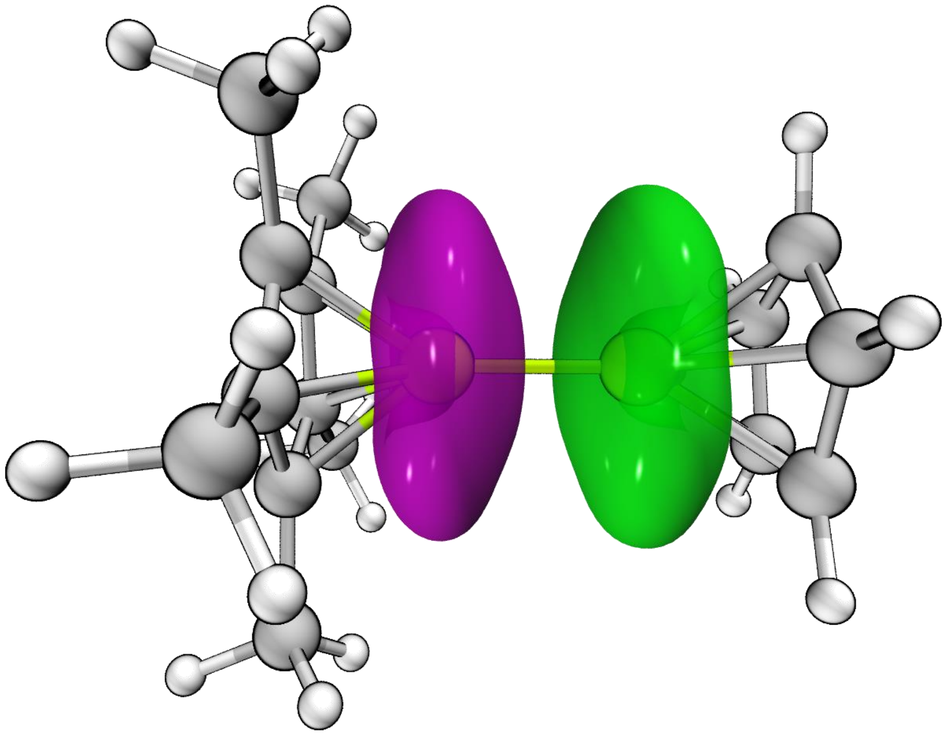

Supplementary Figure 42: NBO for the Be–Be interaction of **2** (0.05 a.u.). Contribution (Be_{Cp}:Be_{Cp*}) to Be-Be bond: 50:50. Wiberg bond index: 0.90. Natural population analysis charges (Be_{Cp}, Be_{Cp*}): 0.86, 0.87.

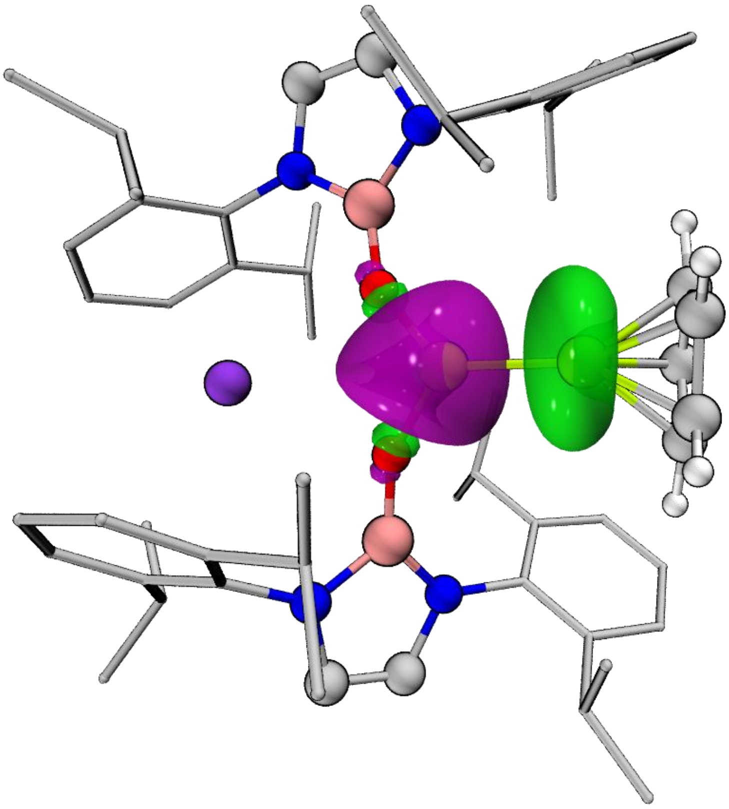

Supplementary Figure 43: NBO for the Be–Be interaction of **2** (0.05 a.u.). Contribution (Be_{Cp}:Be_{NHBO}) to Be-Be bond: 62:38. Wiberg bond index: 0.81. Natural population analysis charges (Be_{Cp}, Be_{NHBO}): 0.64, 1.15.

Natural Energy Decomposition Analysis Calculations

Supplementary Table 8: NEDA data for complex **1**. All values in kcal mol^{-1}. Radical recombination of Be-Be bond is homolytic.

	Complex 1	
	[CpBe]→[BeCp]	Radical Recombination
E_{elstat}	-169.765	-40.524
$E_{orb/ct}$EDA	-272.364	-216.053
E_{core}	213.596	187.655
E_{int}	-228.533	-68.922

Supplementary Table 9: NEDA data for complex **2**. All values in kcal mol^{-1}. Radical recombination of Be-Be bond is homolytic.

	Complex 2		
	[CpBe]→[BeCp*]	[Cp*Be]→[BeCp]	Radical Recombination
E_{elstat}	-166.506	-147.681	-40.379
$E_{orb/ct}$EDA	-274.929	-275.965	-223.654
E_{core}	225.446	199.065	195.823
E_{int}	-215.989	-224.581	-68.21

Supplementary Table 10: NEDA data for complex **3**. All values in kcal mol^{-1}. Radical recombination of Be-Be bond is homolytic.

	Complex 3		
	[CpBe]→[Be(NHBO)$_2$K]	[K(NHBO)$_2$Be]→[BeCp]	Radical Recombination
E_{elstat}	-203.066	-64.235	-69.911
$E_{orb/ct}$EDA	-216.848	-510.96	-282.486
E_{core}	241.376	336.078	282.709
E_{int}	-178.538	-239.117	-69.688

Supplementary Table 11: NEDA data for complex **B**. All values in kcal mol^{-1}. Radical recombination of B-B bond is homolytic. Calculations for the opposite donor-acceptor combination (i.e., [(F)(pin)B→B(pin)]$^-$) collapse.

	Compound B	
	[(pin)B→B(F)(pin)]$^-$	Radical Recombination
E_{elstat}	-117.941	-75.608
$E_{orb/ct}$EDA	-411.471	-520.936
E_{core}	410.891	497.963
E_{int}	-118.521	-98.581

Supplementary Table 12: NEDA data for complex **C**. All values in kcal mol^{-1}. Radical recombination of N-B bond is homolytic. Calculations for the opposite donor-acceptor combination (i.e., $H_3B{\rightarrow}NH_3$) collapse.

	**Compound C**	
	**$H_3N{\rightarrow}BH_3$**	**Radical Recombination**
**E_{elstat}**	-75.659	-223.392
**$E_{orb/ct}$EDA**	-216.432	-532.4
**E_{core}**	246.564	367.755
**E_{int}**	-45.527	-388.038

Energy Decomposition Analysis with Natural Orbitals for Chemical Valence Calculations

Supplementary Table 13: EDA-NOCV data for compounds **2** and **3**. All values in kcal mol^{-1}. Radical fragmentation (0 2,0 –2) of the bond under investigation is homolytic.

	**Compound 2**		**Compound 3**	
	**[CpBe]→[BeCp*]**	**Radical**	**[CpBe]→[Be(NHBO)$_2$K]**	**Radical**
**E_{orb} Total**	-117.62	-61.73	-115.67	-70.08
**E_{orb1}**	-93.49	-57.75	-86.05	-60.52
**E_{orb2}**	-5.88	-1.18	-4.01	-1.25

Supplementary Table 14: EDA-NOCV data for compounds **B** and **C**. All values in kcal mol^{-1}. Radical fragmentation (**B**: 0 2, –1 –2; **C**: +1 2, –1 –2) of the bond under investigation is homolytic.

	Compound **B**		Compound **C**	
	**[(pin)B→B(F)(pin)]$^-$**	Radical	**$H_3N{\rightarrow}BH_3$**	**Radical**
**E_{orb} Total**	-205.05	-142.2	-78.76	-334.64
**E_{orb1}**	-176.93	-101.01	-65.43	-258.93
**E_{orb2}**	-9.39	-3.03	-2.83	-43.23

Supplementary Table 15: EDA-NOCV eigenvalue data for compounds **1**, **3**, and **B** and their respective fragments.

		**Compound 1**	**Compound 3**	**Compound B**
**Total** (α_1+β_1)	**E /** kcal mol^{-1}	-59.7	-60.52	-131.01
	**Eigenvalue**	0.60224	0.64854	0.73067
**Pair α_1**	**E /** kcal mol^{-1}	-29.85	-27.25	-53.24
	**Eigenvalue**	0.30111	0.28546	0.32795
	**% of Total**	49.99833953	44.01578931	44.88346312
**Pair β_1**	**E /** kcal mol^{-1}	-29.85	-33.27	-77.77
	**Eigenvalue**	0.30113	0.36308	0.40272
	**% of Total**	50.00166047	55.98421069	55.11653688
**Difference**		0.001660468	5.984210689	5.116536877

Supplementary Table 16: EDA-NOCV eigenvalue data for selected organic compounds and their respective fragments.

		**$H_3C–CH_3$**	**$F_3C–CH_3$**	**$(C_6F_5)_3C–CH_3$**
**Total** (α_1+β_1)	**E /** kcal mol^{-1}	-229.48	-245.5	-240.24
	**Eigenvalue**	0.931	0.92518	1.02008
**Pair α_1**	**E /** kcal mol^{-1}	-114.74	-110.42	-138.54
	**Eigenvalue**	0.4655	0.42801	0.55771
	**% of Total**	50	46.26235	54.67316
**Pair β_1**	**E /** kcal mol^{-1}	-114.74	-135.08	-101.7
	**Eigenvalue**	0.4655	0.49717	0.46237
	**% of Total**	50	53.73765	45.32684
**Difference**		0	3.737651	4.673163

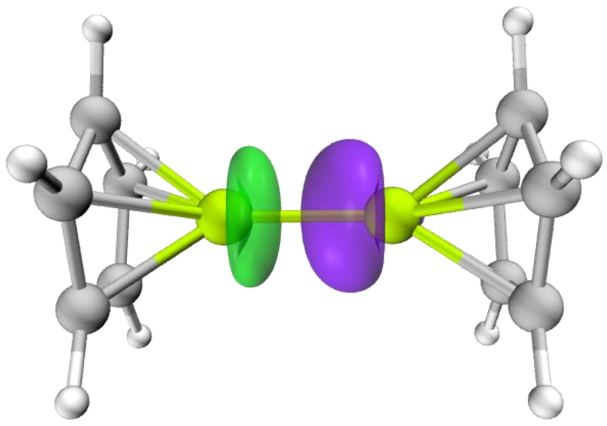

Supplementary Figure 44: Visualisation of EDA-NOCV deformation density. Compound **1**, Pair α_1.

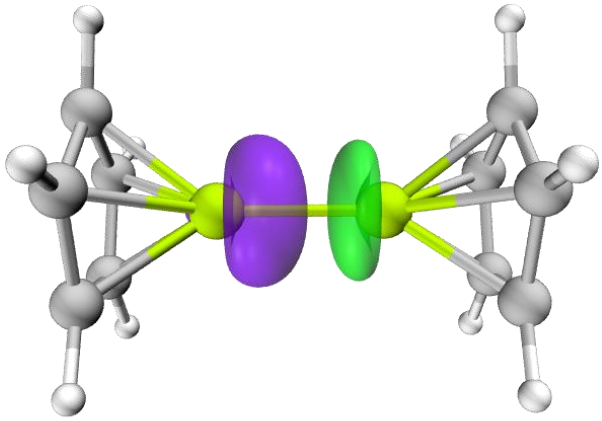

Supplementary Figure 45: Visualisation of EDA-NOCV deformation density. Compound **1**, Pair β_1.

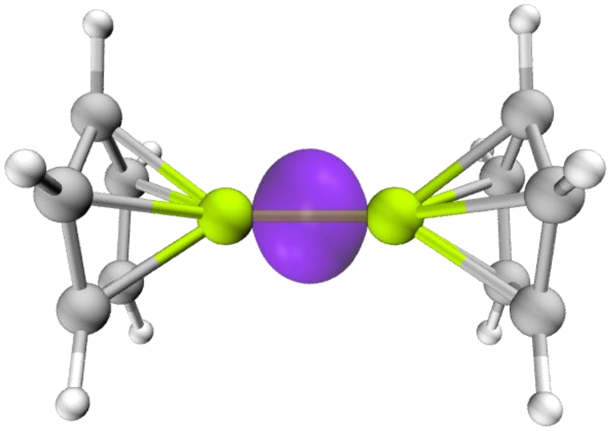

Supplementary Figure 46: Visualisation of EDA-NOCV deformation density. Compound **1**, Total (α_1+β_1).

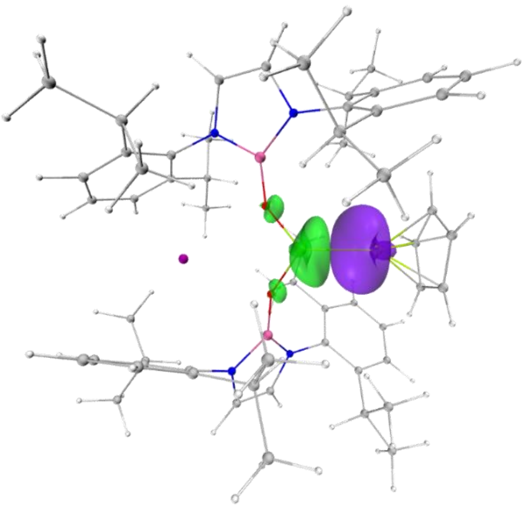

Supplementary Figure 47: Visualisation of EDA-NOCV deformation density. Compound **3**, Pair α_1.

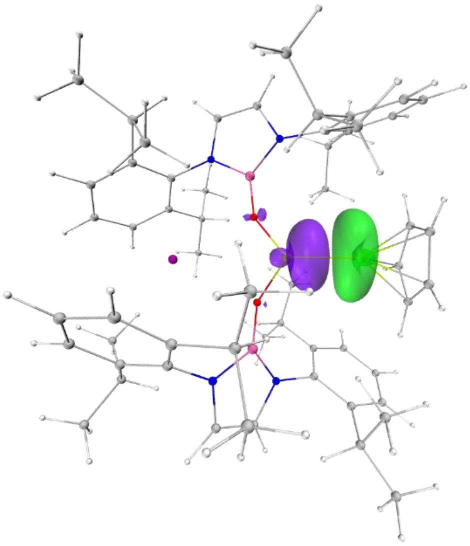

Supplementary Figure 48: Visualisation of EDA-NOCV deformation density. Compound **3**, Pair β_1.

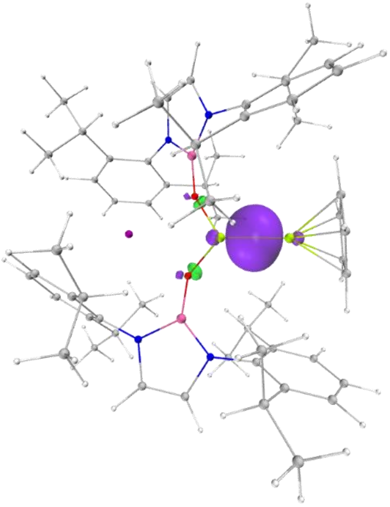

Supplementary Figure 49: Visualisation of EDA-NOCV deformation density. Compound **3**, Total (α_1+β_1).

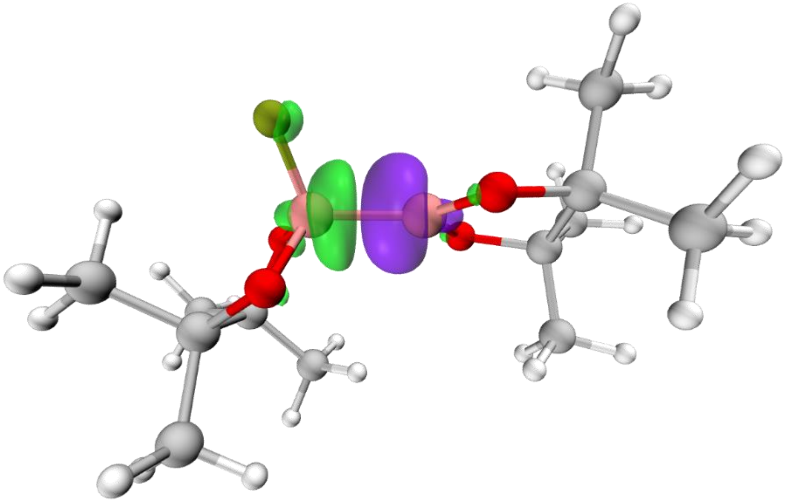

Supplementary Figure 50: Visualisation of EDA-NOCV deformation density. Compound **B**, Pair α_1.

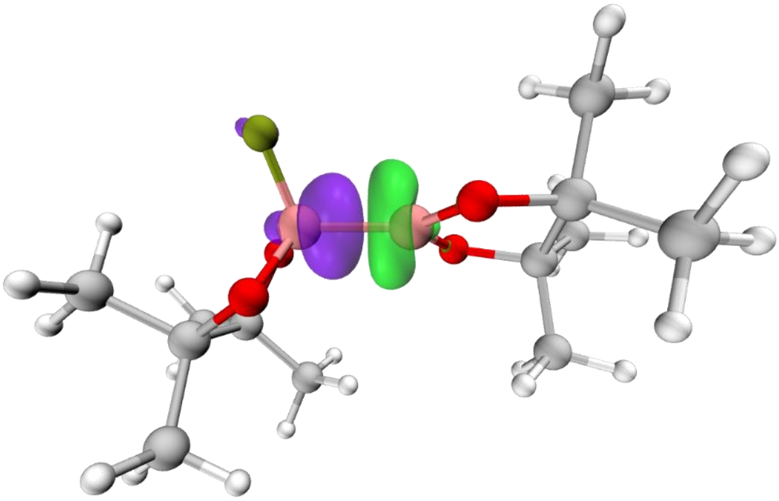

Supplementary Figure 51: Visualisation of EDA-NOCV deformation density. Compound **B**, Pair β_1.

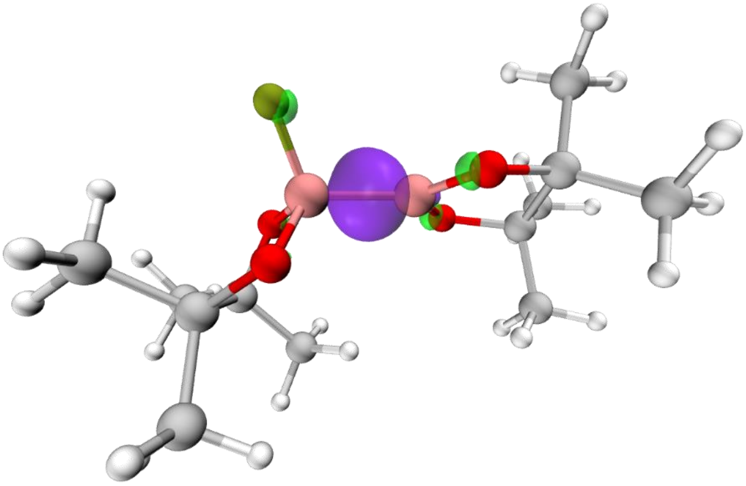

Supplementary Figure 52: Visualisation of EDA-NOCV deformation density. Compound **B**, Total (α_1+β_1).

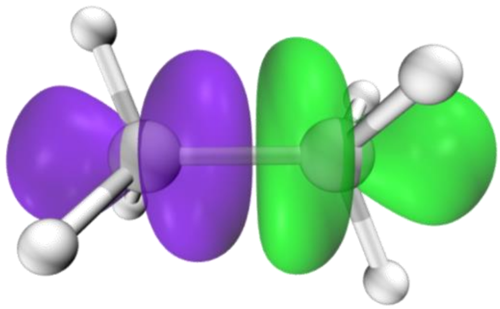

Supplementary Figure 53: Visualisation of EDA-NOCV deformation density. H_3CCH_3, Pair α_1.

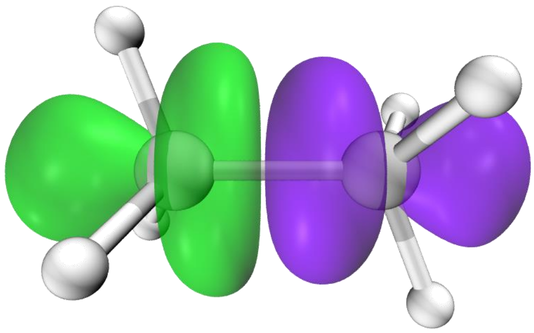

Supplementary Figure 54: Visualisation of EDA-NOCV deformation density. H_3CCH_3, Pair β_1.

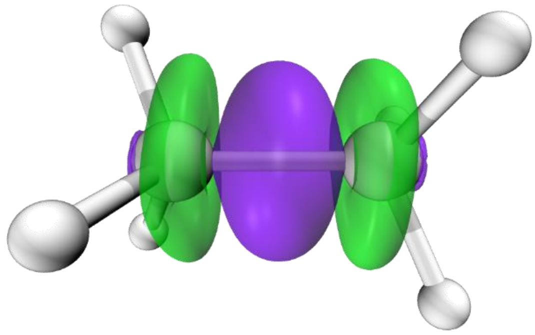

Supplementary Figure 55: Visualisation of EDA-NOCV deformation density. H_3CCH_3, Total (α_1+β_1).

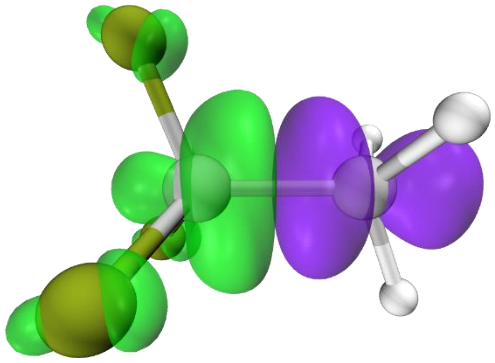

Supplementary Figure 56: Visualisation of EDA-NOCV deformation density. F_3CCH_3, Pair α_1.

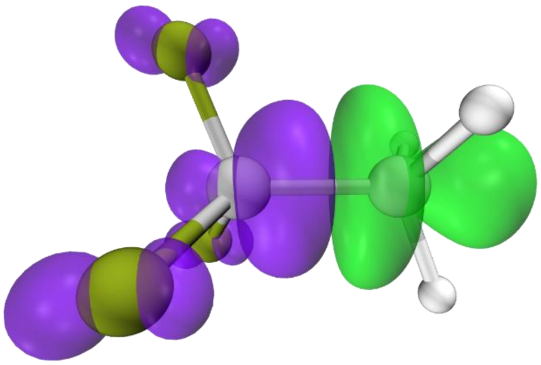

Supplementary Figure 57: Visualisation of EDA-NOCV deformation density. F_3CCH_3, Pair β_1.

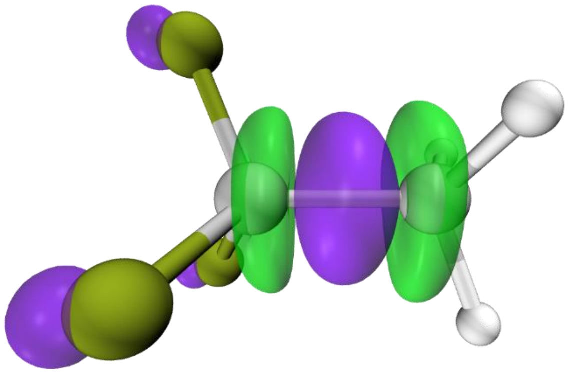

Supplementary Figure 58: Visualisation of EDA-NOCV deformation density. F_3CCH_3, Total (α_1+β_1).

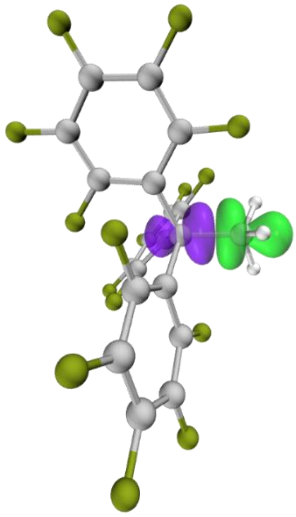

Supplementary Figure 59: Visualisation of EDA-NOCV deformation density. $(C_6F_5)_3CCH_3$, Pair α_1.

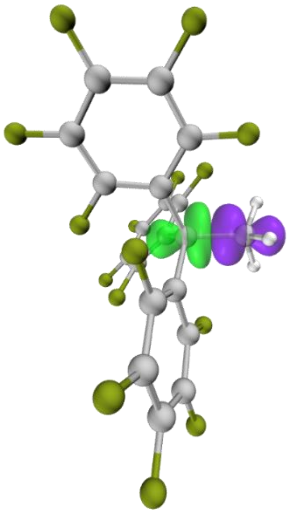

Supplementary Figure 60: Visualisation of EDA-NOCV deformation density. $(C_6F_5)_3CCH_3$, Pair β_1.

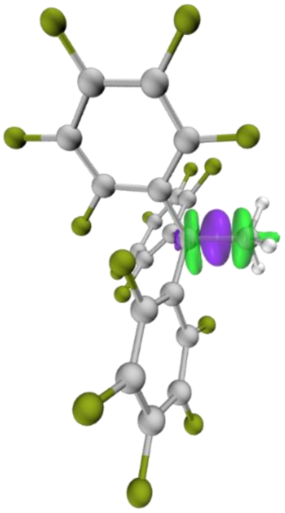

Supplementary Figure 61: Visualisation of EDA-NOCV deformation density. $(C_6F_5)_3CCH_3$, Total (α_1+β_1).

References

1. Segawa, Y., Suzuki, Y., Yamashita, M. & Nozaki, K. Chemistry of Boryllithium: Synthesis, Structure, and Reactivity. *J. Am. Chem. Soc.* **130**, 16069–16079 (2008).
2. Buchanan, J. K. & Plieger, P. G. 9Be nuclear magnetic resonance spectroscopy trends in discrete complexes: An update. *Zeitschrift fur Naturforsch. - Sect. B J. Chem. Sci.* **75**, 459–472 (2020).
3. Plieger, P. G. *et al.* Predicting 9Be nuclear magnetic resonance chemical shielding tensors utilizing density functional theory. *J. Am. Chem. Soc.* **126**, 14651–14658 (2004).
4. Boronski, J. T. *et al.* Inducing Nucleophilic Reactivity at Beryllium with an Aluminyl Ligand. *J. Am. Chem. Soc.* **145**, 4408–4413 (2023).
